# Anticancer Activity, Reduction Mechanism and G-Quadruplex DNA Binding of a Redox-Activated Platinum(IV)–Salphen Complex

**DOI:** 10.3390/ijms232415579

**Published:** 2022-12-08

**Authors:** Vincenzo Vigna, Stefano Scoditti, Angelo Spinello, Gloria Mazzone, Emilia Sicilia

**Affiliations:** 1 Department of Chemistry and Chemical Technologies, Università della Calabria, Via P. Bucci, 87036 Arcavacata di Rende, Italy; 2 Dipartimento di Scienze e Tecnologie Biologiche, Chimiche e Farmaceutiche, Viale delle Scienze, Edificio 17, 90128 Palermo, Italy

**Keywords:** Pt(IV) complexes, G-quadruplex, DFT, MD

## Abstract

Aiming at reducing the unselective cytotoxicity of Pt(II) chemotherapeutics, a great deal of effort has been concentrated into the design of metal-containing drugs with different anticancer mechanisms of action. Inert Pt(IV) prodrugs have been proposed to be a valid alternative as they are activated by reduction directly into the cell releasing active Pt(II) species. On the other hand, a promising strategy for designing metallodrugs is to explore new potential biological targets rather than canonical B-DNA. G-quadruplex nucleic acid, obtained by self-assembly of guanine-rich nucleic acid sequences, has recently been considered an attractive target for anticancer drug design. Therefore, compounds capable of binding and stabilizing this type of DNA structure would be greatly beneficial in anticancer therapy. Here, computational analysis reports the mechanism of action of a recently synthesized Pt(IV)–salphen complex conjugating the inertness of Pt(IV) prodrugs with the ability to bind G-quadruplexes of the corresponding Pt(II) complex. The reduction mechanism of the Pt(IV) complex with a biological reducing agent was investigated in depth by means of DFT, whereas classical MD simulations were carried out to shed light into the binding mechanism of the released Pt(II) complex. The results show that the Pt(IV) prodrug may be reduced by both inner- and outer-sphere mechanisms, and the active Pt(II) complex, as a function of its protonation state, stabilizes the G-quadruplex DNA prevalently, either establishing π-stacking interactions with the terminal G-tetrad or through electrostatic interactions along with H-bonds formation.

## 1. Introduction

The mechanism of action of the most famous and frequently used Pt-based anticancer agent cisplatin has been intensively investigated [1,2,3]. It has been clarified that cisplatin exerts its anticancer activity via multiple mechanisms, although its most prominent and best understood mode of action involves attacking genetic DNA, resulting in transcription dysfunction, translation and other processes ultimately causing tumor cell death. Well-known limitations, such as severe side effects, together with intrinsic or acquired resistance [4,5], associated with the use of cisplatin in clinical practice and its FDA-approved derivatives, nevertheless, have driven the design of new metallodrugs possessing different mechanisms of action [6,7]. One promising strategy for the development of new metal-containing anticancer drugs is to identify novel potential biological targets, such as DNA non-canonical structural arrangements with possible roles in carcinogenic events. G-quadruplex nucleic acids, being very different structurally from the canonical double helix, have recently attracted a great deal of interest as attractive potential therapeutical targets.

Guanine-rich (G-rich) stretches of DNA, first reported by Davies et al. in 1962 [8], have a high propensity to self-associate into planar guanine quartets (G-quartets) to give unusual structures called G-quadruplexes. G-quadruplex (G-Q) structures are formed by stacked flat guanine tetrads (G-tetrads). In each G-tetrad, four guanine bases are arranged in a square plane with Hoogsteen hydrogen bonding, thus creating tetrads stabilized both by π-π stacking interactions, and by the presence of alkali–metal cations (such as Na^+^ and K^+^), which establish electrostatic interactions with the guanine carbonyl groups. Quadruplexes can form from a single nucleic acid sequence in an intramolecular fashion or intermolecularly by conveying together two or more strands. The wide range of topologies that the resulting structures can exhibit have been widely reviewed [9,10].

It has been reported that a number of important regions in the human genome, including oncogene promoters as well as telomeres, adopt G-Q structures, and these regions have been identified as promising targets for anticancer drugs [11,12]. In the case of telomeres, it has been demonstrated that the formation of telomeric G-quadruplexes inhibits telomere elongation by telomerase [13]. This inhibition causes apoptosis of cancer cells, and, since telomerase is overexpressed in the majority of these cells, it is a potential cancer-specific target for avoiding cell death in chemotherapy treatments of tumors. Therefore, a great deal of effort has been devoted to both the identification and the design of small ligands able to interact with and stabilize this secondary DNA structure [14,15].

Although most reported quadruplex DNA binders are based on purely organic templates, it has been shown that metal complexes can also strongly and selectively interact with quadruplex nucleic acids, and the number of reported examples has increased rapidly in recent years [15,16].

Amid the series of metal complexes that bind strongly to quadruplex DNA, one of these is based on metals such as nickel(II), copper(II), and zinc(II) coordinated to salen and salphen tetradentate ligands [17,18]. Additionally, platinum(II)−salphen derivatives as effective binders of quadruplex DNA have been reported [19]. Among the recently synthetized platinum complexes showing good affinity for G-Q DNA, our attention has been attracted by an octahedral Pt(IV)–salphen complex that is an example of a reduction-activated G-Q DNA binder [20].

**Scheme 1:**
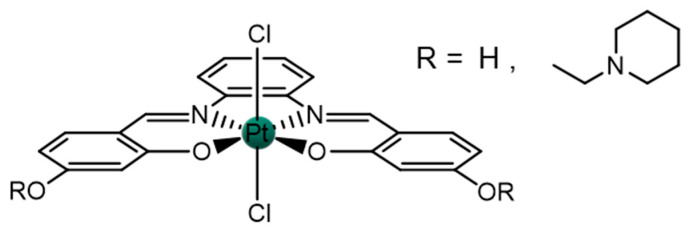
Schematic representation of the investigated **Pt(IV)-Sal**.

Aimed at overcoming typical drawbacks of Pt(II) anticancer agents, six-coordinate Pt(IV) complexes have been proposed as inert prodrugs able to release the corresponding four-coordinate active Pt(II) species upon reduction by cellular reducing agents or by photoactivation [21,22,23]. As Pt(IV) complexes are relatively inert to substitution [24,25,26], reactions with biological nucleophiles are disfavored and the lifetime in biological fluids increases. The octahedral Pt(IV)–salphen complex (**Pt(IV)-Sal**),with two chlorido ligands in the axial position, examined here and shown in Scheme 1, was synthesized as an inert complex able to release by reduction the corresponding Pt(II) analogue (**Pt(II)-Sal**) N,N′-Bis [4-[[1-(2-ethyl)piperidine]oxy]salicylidene]-1,2-phenylenediamine-Pt(II)), which is a good G-Q binder. The affinity of the released **Pt(II)-Sal** complex for G-Q DNA was tested with respect to the G-rich DNA structures *c-myc* and H-Telo in both Na^+^ and K^+^, demonstrating that the examined Pt(II) complex is a good G-Q binder.

In the present paper, by means of quantum-mechanical DFT computations, the propensity of the octahedral **Pt(IV)-Sal** prodrug to be converted by bioreductants such as ascorbic acid (AscH_2_) and glutathione (GSH) into the corresponding square planar **Pt(II)-Sal** G-Q binder was examined. Both inner- and outer-sphere mechanisms were explored. All-atom classical Molecular Dynamics (MD) calculations have been carried out to shed light on the mode of binding of the released Pt(II) complex with G-Q DNA.

## 2. Results

### 2.1. Pt(IV)-Sal Complex Reduction Mechanism

**Scheme 2:**
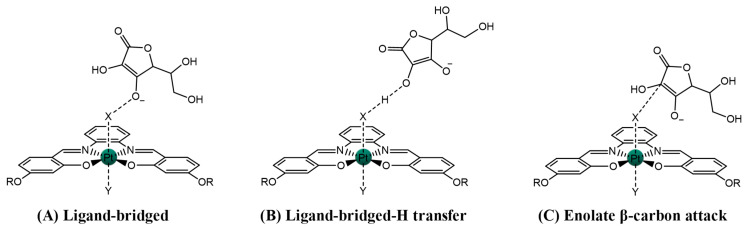
Inner-sphere mechanisms for the reduction of Pt(IV) complexes by AscH^−^ and Cys.

The key step of the mechanism of action of Pt(IV) prodrugs is the reduction, inside the cell, leading to the formation of the corresponding Pt(II) species and the release of the axial ligands. The mechanisms by which the reduction of Pt(IV) complexes can occur are generally classified as inner- and outer-sphere. The main characteristic of the former mechanism is the direct interaction between reacting species, leading to the formation of new bonds, whereas the transfer of the electrons without a direct contact characterizes the latter mechanism. A further distinction among the viable inner-sphere mechanisms, in the presence of AscH_2_ and GSH bioreductants, was proposed by us [27] as (A) ligand-bridged, (B) ligand-bridged H-transfer and (C) enolate β-carbon attack. Mechanism (A) is common to both AscH_2_ and GSH, whereas the (B) and (C) mechanisms are operative when AscH_2_ is the reducing agent. It is worth mentioning that the active form of AscH_2_ is the monoanionic one (AscH^−^), the most abundant at physiological pH (pKa ca. 3.8), and L-Cysteine (Cys) has been used as a model of sulfur-containing bioreductants. As illustrated in Scheme 2, in both the (A) and (B) mechanisms, one of the axial ligands is able to form a bridge between the Pt(IV) center and the reductant facilitates the flow of the electrons. In mechanism (B), however, the electron transfer is accompanied by a proton shift from the reductant to the ligand. Whether the reduction occurs following the (A) or (B) mechanism depends on the identity of the axial ligands. Labile ligands, such as halides, follow mechanism (A), while the release of less labile ligands, such hydroxide and carboxylates, is assisted by a proton transfer as in mechanism (B). Mechanism (C) involves the nucleophilic attack of the enolate β-carbon [28] on the axial ligand, causing the formation of a new bond with the carbon atom and the subsequent detachment.

Concerning the outer-sphere mechanism, for the estimation of the standard redox potential, a decomposition scheme [29], successfully used previously [27,30,31], has been proposed, which assumes that the Pt(IV) prodrug reduction can take place through a two-steps electron transfer including formation of a metastable six-coordinate Pt(III) intermediate.

Both inner- and outer-sphere mechanisms of reduction were investigated for the **Pt(IV)-Sal** complex.

#### 2.1.1. Pt(IV)-Sal Inner-Sphere Reduction Mechanism

**Figure 1:**
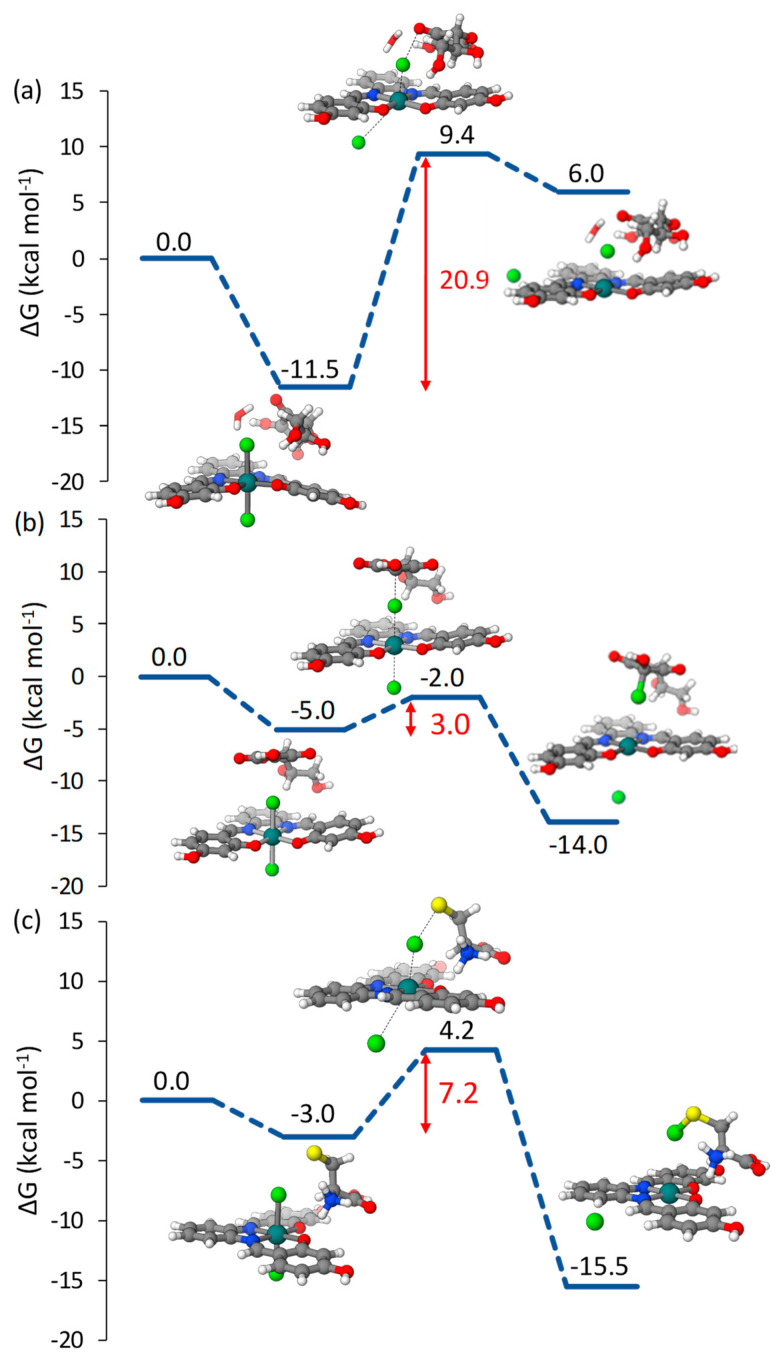
Free energy pathways describing the reduction of the **Pt(IV)-Sal** complex following (**a**) Ligand-bridged and (**b**) β-carbon attack mechanisms in the presence of AscH^−^ and (**c**) ligand-bridged mechanism in the presence of Cys. Relative energies are in kcal mol^−1^ and calculated with respect to the sum of the energies of separated reactants.

The free energy profiles describing the investigated reduction mechanisms of the **Pt(IV)-Sal** complex in the presence of AscH^−^ and Cys are reported in Figure 1, together with the structures of the intercepted stationary points. In order to explore the reduction reactions, the **Pt(IV)-Sal** complex was modelled considering the unsubstituted salphen complex; that is, using R = H as shown in Scheme 2 to reduce the model size and then the computational cost.

Along the pathway describing the ligand-bridged mechanism (A) in the presence of AscH^−^ depicted in Figure 1a, one axial Cl^−^ ligand acts as a bridge, allowing the transfer of two electrons after the exergonic formation, by 11.5 kcal mol^−1^, of the initial adduct stabilized by the hydrogen bonds established between the reacting species. In the transition state, the deprotonated oxygen of the ascorbate interacts with one of the chlorido ligands, two electrons are transferred to the metal center, and, as a consequence, both axial ligands are released. Such rearrangement requires an energetic cost of 20.9 kcal mol^−1^ to occur, resulting in the highly endergonic release of anionic axial ligands, reduced platinum complex and protonated dehydroascorbate products. Such products, indeed, are 6.0 kcal mol^−1^ higher in energy with respect to the zero-reference energy set as the sum of the energies of separated reactants.

The second explored mechanism, displayed in Figure 1b, involves the nucleophilic attack of the enolate β-carbon of ascorbate on one of the chlorido ligands, leading to the formation of a new C-Cl bond and the release of the chloride in the trans position. In the energetically favored adduct formation, the AscH^−^ reductant is oriented to facilitate the nucleophilic attack on the axial ligand. In the transition state, a new C-Cl bond forms, and, simultaneously, the trans chloride detaches. The energy barrier results were very accessible with a value of only 3.0 kcal mol^−1^. The overall reaction was calculated to be exergonic by 14.0 kcal mol^−1^ and led to the release of the **Pt(II)-Sal** complex, one anionic Cl^−^ ligand and the intermediate species in which the second chloride is bound to the β-carbon of the enolate in the geminal position with respect to the OH group. The reported enolate β-carbon attack must be followed by a second step to generate the final product DHA, which was previously investigated in detail [32]. The illustrated results clearly show that when the ascorbate is the reducing agent, the enolate β-carbon attack is preferred over the ligand-bridged mechanism from both kinetic and thermodynamic points of view.

To explore the reduction of the **Pt(IV)-Sal** complex assisted by sulfur-containing compounds, Cys was used as a model, and, according to previous investigations [28,31], the reactive form of cysteine is the zwitterionic one that becomes even more active when the proton migrates from sulfur to one of the carboxylate oxygen atoms. The reaction mechanism was the ligand-bridging (A) type with the formation of a S-Cl bond enabling the electron flow and causing the release of the ligand at the trans position (Scheme 2).

The calculated free energy profile is shown in Figure 1c, where the transition state allowing the formation of the S-Cl bridge bond and the simultaneous release of the trans anionic Cl ligand lies 7.2 kcal mol^−1^ above the minimum leading to it that, in turn, is stabilized by 3.0 kcal mol^−1^ with respect to reactants. The final products, whose formation was exergonic by 15.5 kcal mol^−1^, were the corresponding **Pt(II)-Sal** complex and the sulfenylchloride formed with the released chlorido bridging ligand.

In order to check whether the inclusion of the two ethyl-piperidine moieties had an influence on the energetics of the reduction process, the minima and transition state along the most favorable pathway, β-carbon attack in the presence of ascorbate, was explored considering the complete structure of the complex (see Scheme 1). The calculated free energy profile is reported in Figure S1 of the Supplementary Materials (SM). The height of the energy barrier that is necessary to overcome for the reduction to occur was calculated to be 4.4 kcal mol^−1^, close to the value calculated for the simplified form of the complex. The first formed adduct was stabilized by 3.1 kcal mol^−1^ with respect to the zero-reference energy and the products, including the active **Pt(II)-Sal** complex, were 13.1 kcal mol^−1^ below the same zero-reference energy reactants.

This in silico analysis confirms that ascorbate and cysteine are able to efficiently reduce **Pt(IV)-Sal** through inner-sphere mechanisms, the reduction reactions being highly exergonic with very accessible energy barriers, particularly when the ascorbate is the reductant.

#### 2.1.2. Pt(IV)-Sal Outer-Sphere Reduction

**Scheme 3:**
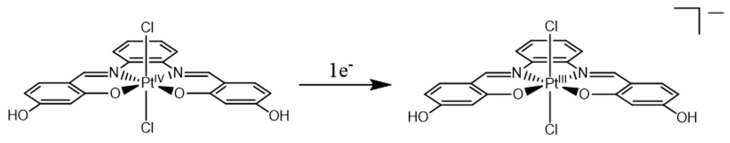
First step of the proposed decomposition scheme for the first electron transfer.

The redox behavior of Pt(IV) prodrugs is routinely investigated using electrochemical approaches, mainly by cyclic voltammetry. However, the standard redox potential cannot be obtained as the mid-point potential from a typical cyclic voltammogram of a chemically reversible process, as the Pt(IV) reduction is irreversible. Both electrochemical and chemical events, indeed, are involved, and the peak potential of the irreversible cathodic response is used to approximately measure the propensity to be reduced of Pt(IV) complexes. Baik et al. carried out a series of experiments that demonstrated that electron transfer and cleavage of the bonds with the leaving ligands occur in a stepwise fashion, involving the formation of a metastable six-coordinate Pt(III) intermediate as a consequence of the first electron addition. The two-electron reduction process was demonstrated to be dominated by the more difficult one-electron first reduction, whereas the second one-electron transfer corresponds to a more positive reduction potential not influencing the value of the total reduction potential [29]. The proposed decomposition scheme allows the calculation of the reduction potential, considering only the free energy change that accompanies the first one-electron transfer leading to the formation of the six-coordinate Pt(III) complex, as shown in Scheme 3. The reduction potential of **Pt(IV)-Sal** was estimated using the following formula: E° = −ΔG_(sol)_ − E_ref_, where ΔG_(sol)_ is the Gibbs free energy change in solution accompanying the one-electron transfer to the Pt(IV) complex and E_ref_ is the absolute potential of the standard electrode used as a reference. Additionally, 4.43 V is the value used here for E_ref_, the absolute potential of the standard hydrogen electrode (SHE) in water. The value of the calculated redox potential is 0.162 eV. The experimental redox potential is similar to that of the cisplatin Pt(IV) derivative *cis*-[PtCl_4_(NH_3_)_2_]. The reduction peak potential of such complex has been measured to be −0.204 eV [33] and was previously calculated by us to be 0.374 eV, adopting the Baik et al. decomposition scheme [27]. The value of 0.162 eV calculated for **Pt(IV)-Sal** by us using the same approach favorably compares with that calculated for the *cis*-[PtCl_4_(NH_3_)_2_] complex because peak potentials are significantly more negative than the normal potentials. Therefore, according to the reported trends in the propensity of Pt(IV) complexes to be reduced by an outer-sphere mechanism as a function of the axial ligand identity [27,34], the **Pt(IV)-Sal** complex may also be reduced following an outer-sphere mechanism.

When the Pt(IV) complex is reduced, the formed **Pt(II)-Sal** can exert its anticancer activity by binding to G-Q DNA.

### 2.2. Pt(IV)-Sal Complex Binding

To cause the inhibition of telomerase and alter the expression of oncogenes, G-quadruplexes, as illustrated above, have to be stabilized. Numerous organic planar small molecules have been studied, as they show many of the characteristics increasing the affinity for G-quadruplexes, such as (i) a π-delocalized system, which assists the stacking of the G-tetrad; (ii) a positive or partial positive charge, which interacts with the O6 of the guanines oriented toward the center of the G-tetrads; (iii) positively charged substituents able to interact with the negatively charged phosphate backbone; and (iv) a surface area similar to the G-tetrads justifying a larger affinity for the G-quadruplex than regular DNA. Nevertheless, even if most of the reported G-quadruplex binding molecules are purely organic structures, metal complexes offer a broad range of electronic and structural properties that can be exploited for designing new drugs able to interact with G-tetrads.

#### 2.2.1. Pt(II)-Sal Binding Interactions

**Scheme 4:**
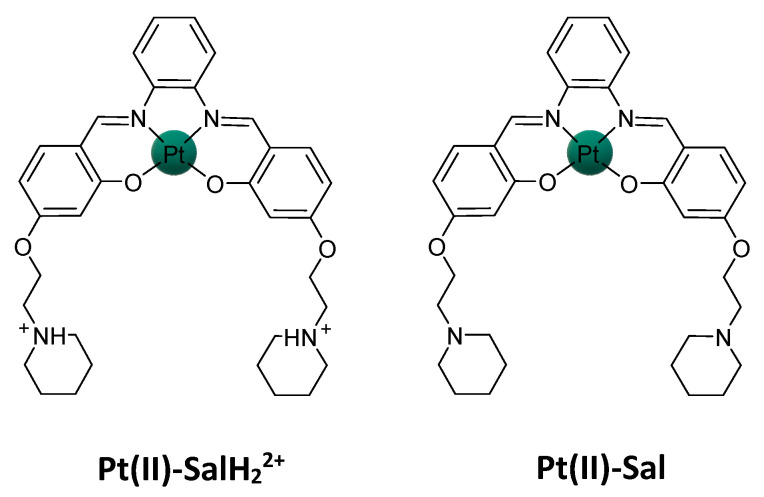
Structures of the two protonation states of **Pt(II)-Sal** considered in MD simulations.

In order to shed light on the binding mechanism of **Pt(II)-Sal** towards G-Q DNA, all-atom classical MD simulations were performed. The parallel G-Q conformation from the promoter region of the oncogene *c-myc* (PDB ID: 1XAV) [35] was selected to investigate the Pt(II) complex binding, since it is the quadruplex conformation reported in previous experimental settings [19,36]. At physiological pH, the side chains of the complex are most likely positively charged (see details in Materials and Methods section) due to the protonation of the piperidine’s nitrogen atoms (Scheme 4).

Therefore, a 300 ns long MD simulation (MD1) of the protonated **Pt(II)-Sal**, hereafter called **Pt(II)-SalH_2_^2+^**, was carried out. Moreover, an additional simulation (MD2) for the deprotonated complex (Scheme 4) was performed using the same starting geometry, aiming to estimate the effective influence of the protonation on the complex affinity towards this G-Q conformation.

**Figure 2:**
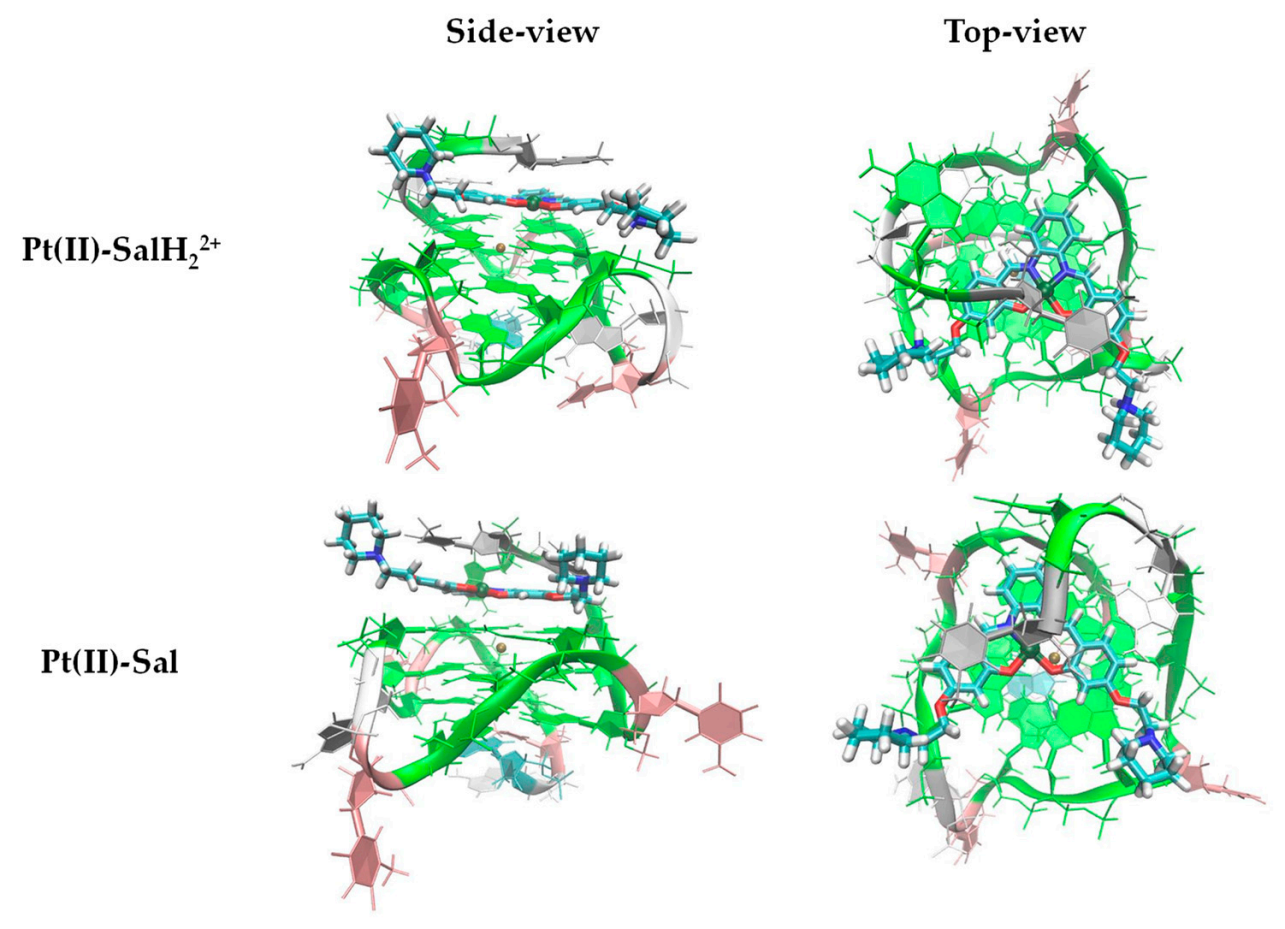
Side and top views of the binding poses for **Pt(II)-SalH_2_^2+^** and **Pt(II)-Sal** interacting with the G-quadruplex structure of *c-myc*. Complexes are shown in licorice style; guanines are highlighted in green.

The DNA-binding of the investigated Pt(II) complex is mainly due to π-stacking interactions between its planar aromatic moiety and the terminal G-tetrad of *c-myc*. Representative structures obtained by RMSD-based clustering from the MD trajectories, showing the interaction of both **Pt(II)-SalH_2_^2+^** and **Pt(II)-Sal** with the G-Q, are displayed in Figure 2. In order to better highlight the π-π interactions, an additional figure is provided in the SM (Figure S2). Concerning the binding of **Pt(II)-SalH_2_^2+^**, stacking might be facilitated by H-bonds (see Table S1 in the SM) and electrostatic attraction established by the positively charged piperidine’s nitrogen atoms and the negatively charged phosphate backbone of the G-Q.

The all-atom RMSD increased in both MD1 and MD2 (Figure S3 black line), while the RMSD of the guanine atoms (red line) remained stable, suggesting that all the significant variations are connected to the quadruplex flexible loops and flanking bases.

**Figure 3:**
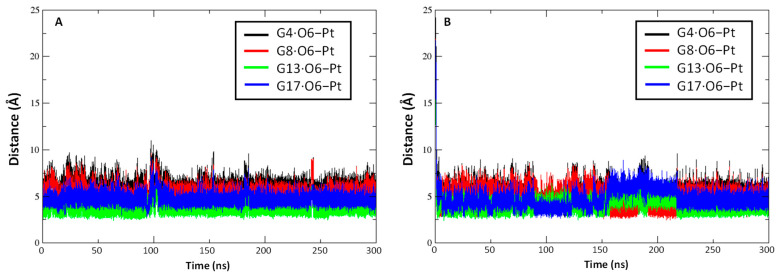
Distance (Å) between Pt and O6 atoms of the four guanines in the 3′ quartet for (**A**) MD1 and (**B**) MD2 simulations.

The distance between the Pt atom and the O6 carbonyl atoms of the four guanines of the terminal G-tetrad along the trajectories has been plotted in Figure 3. In the case of MD1 simulation, the binding pose appeared to be stable during the whole simulation time and the metal atom was almost aligned with the center of the potassium channel, as already observed for analogous salphen-based metal complexes [37,38]. Moreover, in MD2, the neutral complex, after a few initial nanoseconds, also reached a stable binding pose that was maintained during the whole 300 ns simulation.

Binding free energies (ΔG_b_) of **Pt(II)-SalH_2_^2+^** and **Pt(II)-Sal** complexes were calculated by means of the MM-GBSA approach [39] and are included in Table S2. Interestingly, MD2 showed a lower ΔG_b_ of 2.1 kcal mol^−1^ in comparison with the higher binding energy value of −11.8 kcal mol^−1^ obtained for the charged **Pt(II)-SalH_2_^2+^**. This striking difference could be related to the absence, in the neutral form, of key electrostatic interactions established by the positively charged nitrogen atoms of the piperidine side chains that, interacting with the surrounding phosphate groups, increase the binding affinity of the complex. In addition, hydrogen-bond (H-bond) interactions could play a role in increasing the binding energy of the protonated complex with respect to the deprotonated one. In particular, persistent H-bonds, formed between the two piperidine nitrogen atoms and the phosphate groups of the G-Q, were detected in MD1, contributing to the total ΔG_b_ of compound **Pt(II)-SalH_2_^2+^** (Table S1 of the SM). Thus, it can be stated that positive charges and H-bonds clearly favor the affinity of the Pt-complex towards the binding site.

To further verify the reliability of our results, we performed and analyzed an additional 300 ns long replica of the positively charged species **Pt(II)-SalH_2_^2+^** (MD1_rep), which displayed improved binding properties. Here, **Pt(II)-SalH_2_^2+^**, after a few nanoseconds, found a stable binding pose, similarly to MD1, showing a close ΔG_b_ value of −13.2 kcal mol^−1^. This small variation may result from the change of the complex orientation with respect to the G-tetrad, which might alter the drug secondary interaction with DNA (see Figure S4).

#### 2.2.2. Pt(II)-SalH_2_^2+^ Targeting Binding Site

To the best of our knowledge, unbiased classical MD simulations aiming at describing the process by which the drug–target binding occurs are only few [40,41,42,43]. Most of the studies often focus on a ligand that already interacts or is near the binding site, using molecular docking or manual addition of the drug on the binding pocket to generate the pose used as a starting point. Indeed, long simulation times are commonly required, implying a considerable computational cost and complexity, especially for very big targets [40].

**Figure 4:**
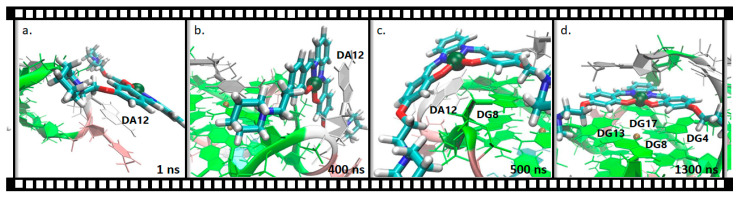
Representative snapshots extracted from 1.5 μs long MD run at (**a**) 1, (**b**) 400, (**c**) 500 and (**d**) 1300 ns, respectively.

Thus, to test the complex capacity of targeting the G-tetrad binding site, we ran an additional MD simulation starting with the **Pt(II)-SalH_2_^2+^** complex, far from the terminal G-tetrad, by 25 Å with respect to its center of mass (Figure S5). The reproduction of the molecular recognition process, using unbiased classical all-atom MD, required a major effort in terms of simulation time and computational cost. In particular, the MD simulation was carried out for 1.5 μs to allow the Pt-complex to correctly recognize and interact with the binding site. The molecular recognition process of **Pt(II)-SalH_2_^2+^**, during the 1.5 μs-long trajectory, is reported as a supplementary movie file (Movie S1). From this simulation, it is possible to extract key information about the secondary interactions that favor the recognition process. As shown in Figure 4, which reports four snapshots extracted from the trajectory, the primary interaction of the drug with the target occurred almost immediately (1 ns) with an adenine (DA12) present in the G-Q structure. This **Pt(II)-SalH_2_^2+^**-DA12 interaction anchors the complex to the G-Q target. When this interaction was established, **Pt(II)-SalH_2_^2+^** continued to sample other conformations, allowing its positively charged ethyl-piperidine moieties to interact with neighboring phosphate groups. As reported in Figure 4b, at 400 ns DA12 rotated, dragging behind the Pt(II) complex that, as shown in the frame extracted at 500 ns (Figure 4c), in this new position interacted with both the adenine nucleobase and a guanine (DG8) of the terminal G-tetrad. This interaction continued for most of the dynamics until, at about 1300 ns (Figure 4d), the complex finally recognized the binding site and the interaction with the nucleobase DA12 broke down.

We additionally used the last 150 ns of the MD simulation to estimate the binding affinity using the MM-GBSA approach to compare it with the results reported above. Remarkably, a ΔG_b_ value of −17.1 kcal mol^−1^ was obtained, higher than all the other simulation results. In fact, a structural comparison with the MD1 binding pose (see Figure S6 in the SM) reveals that **Pt(II)-SalH_2_^2+^** is oriented differently with respect to the other dynamics. This may be due to a better stacking of the complex on the surface of the terminal G-Q quartet, since the complex has more time to properly fit into the binding site using this unbiased technique.

Overall, our results strongly indicate that, together with π-π stacking interactions, recognition and binding of G-Q by **Pt(II)-SalH_2_^2+^** are driven by H-bonds, strong electrostatic attraction between the positively charged piperidine groups and the negatively charged phosphate backbone of the G-Q, allowing the metal complex to easily approach the biomolecule binding site. Moreover, the importance of properly selecting the most reliable protonation state for the investigated ligand is strongly pointed out, since it might profoundly impact the molecular recognition process [44].

## 3. Materials and Methods

All molecular geometry optimizations were carried out with the Gaussian16 software package [45], at the density functional level of theory, employing the B3LYP hybrid exchange–correlation functional [46,47]. Grimme dispersion corrections for non-bonding interactions have been included using the atom pairwise additive scheme denoted as D3 [48]. The relativistic compact Stuttgart/Dresden (SDD) effective core potential and the corresponding split valence basis set were used to describe platinum atoms [49]. The triple-quality basis sets 6–311+G* of Pople et al. for the atoms directly involved in the process were used, while to reduce the computational effort, for peripheral atoms, the 6–311G** basis sets were employed. In addition, the structure of the complex was simplified by removing some peripheral substituents that are irrelevant to the final result in terms of potential energy profile. Optimizations were carried out in implicit water (ε = 78.4) using the PCM continuum solvation model as implemented in Gaussian 16 [50,51].

To confirm their nature as minima or transition states, the located stationary points were checked with vibrational analysis at the same level of theory. The IRC (intrinsic reaction coordinate) analysis was used to check that the transition states involved were properly connected to the minima [52]. The same level of theory was used to calculate both zero-point energy (ZPE) and Gibbs free energy corrections. The Cartesian coordinates of all the optimized structures have been provided in the Supplementary Materials.

Molecular dynamics simulations were performed in order to study the interaction of **Pt(II)-Sal** with G-Q, using a parallel *c-myc* G-Q (PDB ID 1XAV) as a model [35]. The most favorable protonation state of the complex was evaluated using the Epik software [53]. Then, **Pt(II)-Sal**, in its two different protonation states (**Pt(II)-Sal** and **Pt(II)-SalH_2_^2+^**), was manually placed in proximity of the binding site, except for the so-called recognition dynamics, for which the drug was added in a position far from the binding site, in order to evaluate its ability to target the binding site. The platinum metal center was parametrized (detailed information is reported in the SM) by using both Gaussian 16 and MCPB.py [54] in the Amber 16 package [55]. Geometry optimization and frequency calculations were carried out using Gaussian 16 for the metal-center-created model using SDD effective core potential and split valence basis set to describe platinum atom and 6-311G** basis set for the rest of the atoms. For the charges, Gaussian 16 was used to calculate the Merz–Kollman ESP charge of the created metal center model. MCPB.py was, then, used to perform RESP charge fitting. Topology and coordinate files were generated by means of the command-line program tleap of Amber 16 using the standard DNA.bsc1 [56] and gaff force fields [57], together with the newly generated parameters for the metal center. A TIP3P solvation model [58,59,60] was used to construct an octahedral box of water around the G-Q/**Pt(II)-Sal** complex with a 14 Å buffer distance. Sodium ions were added to neutralize the system. The system was relaxed prior to the molecular dynamics by 1000 minimization steps with a cutoff distance of 15.0 Å and a constant volume periodic boundaries. The G-Q and **Pt(II)-Sal** were held fixed during this initial minimization by using a force constant of 500 kcal mol^−1^. This was followed by 2500 minimization steps for the whole system, including the DNA, with the same cutoff distance and constant volume periodic boundaries. The system was, then, heated from 0 K to 300 K over 10,000 steps for a total of 20 ps. The SHAKE algorithm was implemented to constrain bonds involving hydrogen. During the heating, the DNA and **Pt(II)-Sal** were weakly restrained by a force constant of 10 kcal mol^−1^ while keeping constant volume periodic boundaries and the same cutoff distance. Equilibration, followed by production of molecular dynamics for 300 ns at 300 K, were run under similar conditions with a 2 fs interval with no restraints on DNA, except for the recognition, for which a 1500 ns dynamic was carried out. The SHAKE algorithm, to constrain bonds involving hydrogen, and the cutoff distance of 15.0 angstroms, were all maintained. A constant pressure periodic boundary with an average pressure of 1 atm and an isotropic position scaling with a relaxation time of 2 ps were used.

For evaluating the relative binding free energy between the different protonation states of **Pt(II)-Sal** and the G-Q, the MM-GBSA method, as implemented by MMPBSA.py [61] script in the Amber 16 package, was used, evaluating the whole production trajectory over the last 150 ns of each dynamic. Cpptraj [62] in the Amber 16 package was used to generate the data about the RMSD, distances and H-bond. The VMD program was used to display the dynamics trajectory [63].

## 4. Conclusions

In the present paper, the outcomes of a detailed computational investigation of the anticancer mechanism of action of a new Pt(IV)–Salphen complex are shown, having two chlorido ligands in axial positions, able to interact, in its reduced form, with G-quadruplex DNA. Both inner- and outer-sphere reduction mechanisms have been quantum-mechanically explored in presence of ascorbic acid and cysteine as a model of sulfur-reducing agents. This in silico analysis confirms that ascorbate and cysteine are able to efficiently reduce the studied complex through inner-sphere mechanisms, the reduction reactions being highly exergonic with very accessible energy barriers, particularly when the ascorbate is the reductant. On the basis of the value of the estimated reduction potential and the propensity trend of Pt(IV) complexes to be reduced, it appears that the complex can be also reduced by an outer-sphere mechanism. Classical MD simulations have been performed to clarify the G-Q binding mechanism using the parallel G-quadruplex conformation from the promoter region of the oncogene *c-myc*. Due to the possible protonation of the side-chain piperidine nitrogen atoms, the interaction with G-Q of both neutral and charged forms of the Pt(II) complex was examined. The results show that the DNA-binding of the neutral Pt(II) complex is mainly due to π-stacking interactions between its planar aromatic moiety and the terminal G-tetrad of *c-myc*. When the protonated form is taken into consideration, electrostatic interactions, along with the possibility of forming H-bonds, increase the ability of the complex to recognize and stably bind the *c-myc* G-Q target. This last result highlights the importance of properly selecting the most reliable protonation state for the investigated ligand, since it might significantly influence the molecular recognition process.

## Data Availability

Not applicable.

## Supplementary Materials

The following supporting information can be downloaded at: https://www.mdpi.com/article/10.3390/ijms232415579/s1, Figure S1. Free energy pathways describing the reduction considering the complete structure of the complex **Pt(IV)-Sal** complex following β-carbon attack mechanisms in the presence of AscH^−^. Relative energies are in kcal mol^−1^ and calculated with respect to the sum of the energies of separated reactants; Figure S2. Improved view of π-π interactions of **Pt(II)-SalH_2_^2+^** and **Pt(II)-Sal** with the G-Q; Figure S3. RMSD plot of MD1 (A) and MD2 (B); Figure S4. Structural comparison between the binding poses of the complexes in MD1 and MD1_rep; Figure S5. Starting point of the targeting 1.5 μs MD run, MD1_targeting; Figure S6. Structural comparison between the binding poses of **Pt(II)-SalH_2_^2+^** in MD1 and MD1_targeting; Table S1. H-bond showing a persistence higher than 10% within MD1; Table S2. Contributions to the MM-GBSA binding free energy for the Q-G-complex adducts. Van der Waals, electrostatic, polar and non-polar contributions to the solvation free energy, total gas phase and solvation binding energy, resulting MM-GBSA binding energy and the estimation of the entropy term by quasi-harmonic analysis are reported; developed parameters for **Pt(II)-SalH_2_^2+^**; developed parameter for **Pt(II)-Sal**; Cartesian coordinates. Movie S1: molecular recognition process of **Pt(II)-SalH_2_^2+^**, during the 1.5 μs-long trajectory.

## References

[1] Kelland L. (2007). The Resurgence of Platinum-Based Cancer Chemotherapy. Nat. Rev. Cancer.

[2] Reedijk J. (2003). New Clues for Platinum Antitumor Chemistry: Kinetically Controlled Metal Binding to DNA. Proc. Natl. Acad. Sci. USA.

[3] Jung Y., Lippard S.J. (2007). Direct Cellular Responses to Platinum-Induced DNA Damage. Chem. Rev..

[4] Heffeter P., Jungwirth U., Jakupec M., Hartinger C., Galanski M.S., Elbling L., Micksche M., Keppler B., Berger W. (2008). Resistance against Novel Anticancer Metal Compounds: Differences and Similarities. Drug Resist. Updates.

[5] Yao X., Panichpisal K., Kurtzman N., Nugent K. (2007). Cisplatin Nephrotoxicity: A Review. Am. J. Med. Sci..

[6] Bruijnincx P.C., Sadler P.J. (2008). New Trends for Metal Complexes with Anticancer Activity. Curr. Opin. Chem. Biol..

[7] Ho Y.-P., Au-Yeung S.C.F., To K.K.W. (2003). Platinum-Based Anticancer Agents: Innovative Design Strategies and Biological Perspectives. Med. Res. Rev..

[8] Gellert M., Lipsett M.N., Davies D.R. (1962). Helix Formation by Guanylic Acid. Proc. Natl. Acad. Sci. USA.

[9] Patel D.J., Phan A.T., Kuryavyi V. (2007). Human Telomere, Oncogenic Promoter and 5’-UTR G-Quadruplexes: Diverse Higher Order DNA and RNA Targets for Cancer Therapeutics. Nucleic Acids Res..

[10] Burge S., Parkinson G.N., Hazel P., Todd A.K., Neidle S. (2006). Quadruplex DNA: Sequence, Topology and Structure. Nucleic Acids Res..

[11] Hänsel-Hertsch R., di Antonio M., Balasubramanian S. (2017). DNA G-Quadruplexes in the Human Genome: Detection, Functions and Therapeutic Potential. Nat. Rev. Mol. Cell Biol..

[12] Müller S., Rodriguez R. (2014). G-Quadruplex Interacting Small Molecules and Drugs: From Bench toward Bedside. Expert Rev. Clin. Pharmacol..

[13] Fletcher T.M., Sun D., Salazar M., Hurley L.H. (1998). Effect of DNA Secondary Structure on Human Telomerase Activity. Biochemistry.

[14] Campbell N., Collie G.W., Neidle S. (2012). Crystallography of DNA and RNA G-Quadruplex Nucleic Acids and Their Ligand Complexes. Curr. Protoc. Nucleic Acid Chem..

[15] Georgiades S.N., Abd Karim N.H., Suntharalingam K., Vilar R. (2010). Interaction of Metal Complexes with G-Quadruplex DNA. Angew. Chem. Int. Ed..

[16] Campbell N.H., Karim N.H.A., Parkinson G.N., Gunaratnam M., Petrucci V., Todd A.K., Vilar R., Neidle S. (2012). Molecular Basis of Structure–Activity Relationships between Salphen Metal Complexes and Human Telomeric DNA Quadruplexes. J. Med. Chem..

[17] Reed J.E., Arnal A.A., Neidle S., Vilar R. (2006). Stabilization of G-Quadruplex DNA and Inhibition of Telomerase Activity by Square-Planar Nickel(II) Complexes. J. Am. Chem. Soc..

[18] Arola-Arnal A., Benet-Buchholz J., Neidle S., Vilar R. (2008). Effects of Metal Coordination Geometry on Stabilization of Human Telomeric Quadruplex DNA by Square-Planar and Square-Pyramidal Metal Complexes. Inorg. Chem..

[19] Wu P., Ma D.-L., Leung C.-H., Yan S.-C., Zhu N., Abagyan R., Che C.-M. (2009). Stabilization of G-Quadruplex DNA with Platinum(II) Schiff Base Complexes: Luminescent Probe and Down-Regulation of c- Myc Oncogene Expression. Chem. A Eur. J..

[20] Bandeira S., Gonzalez-Garcia J., Pensa E., Albrecht T., Vilar R. (2018). A Redox-Activated G-Quadruplex DNA Binder Based on a Platinum(IV)-Salphen Complex. Angew. Chem. Int. Ed..

[21] Butler J.S., Sadler P.J. (2013). Targeted Delivery of Platinum-Based Anticancer Complexes. Curr. Opin. Chem. Biol..

[22] Pathak R.K., Marrache S., Choi J.H., Berding T.B., Dhar S. (2014). The Prodrug Platin- A: Simultaneous Release of Cisplatin and Aspirin. Angew. Chem. Int. Ed..

[23] Graf N., Lippard S.J. (2012). Redox Activation of Metal-Based Prodrugs as a Strategy for Drug Delivery. Adv. Drug Deliv. Rev..

[24] Bradáč O., Zimmermann T., Burda J.v. (2013). Can Satraplatin Be Hydrated before the Reduction Process Occurs? The DFT Computational Study. J. Mol. Model..

[25] Ritacco I., Mazzone G., Russo N., Sicilia E. (2016). Investigation of the Inertness to Hydrolysis of Platinum(IV) Prodrugs. Inorg. Chem..

[26] Wexselblatt E., Yavin E., Gibson D. (2013). Platinum(IV) Prodrugs with Haloacetato Ligands in the Axial Positions Can Undergo Hydrolysis under Biologically Relevant Conditions. Angew. Chem. Int. Ed..

[27] Dabbish E., Ponte F., Russo N., Sicilia E. (2019). Antitumor Platinium(IV) Prodrugs: A Systematic Computational Exploration of Their Reduction Mechanism by L-Ascorbic Acid. Inorg. Chem..

[28] Ejehi Z., Ariafard A. (2017). A Computational Mechanistic Investigation into the Reduction of Pt(IV) Prodrugs with Two Axial Chlorides by Biological Reductants. Chem. Commun..

[29] McCormick M.C., Keijzer K., Polavarapu A., Schultz F.A., Baik M.-H. (2014). Understanding Intrinsically Irreversible, Non-Nernstian, Two-Electron Redox Processes: A Combined Experimental and Computational Study of the Electrochemical Activation of Platinum(IV) Antitumor Prodrugs. J. Am. Chem. Soc..

[30] Scoditti S., Mazzone G., Sanna N., Sicilia E. (2022). Computational Exploration of the Synergistic Anticancer Effect of a Multi-Action Ru(II)–Pt(IV) Conjugate. Inorg. Chem..

[31] Scoditti S., Vigna V., Dabbish E., Sicilia E. (2021). Iodido Equatorial Ligands Influence on the Mechanism of Action of Pt(IV) and Pt(II) Anti-cancer Complexes: A DFT Computational Study. J. Comput. Chem..

[32] Ponte F., Russo N., Sicilia E. (2018). Insights from Computations on the Mechanism of Reduction by Ascorbic Acid of Pt IV Prodrugs with Asplatin and Its Chlorido and Bromido Analogues as Model Systems. Chem. A Eur. J..

[33] Gramatica P., Papa E., Luini M., Monti E., Gariboldi M.B., Ravera M., Gabano E., Gaviglio L., Osella D. (2010). Antiproliferative Pt(IV) Complexes: Synthesis, Biological Activity, and Quantitative Structure–Activity Relationship Modeling. JBIC J. Biol. Inorg. Chem..

[34] Wexselblatt E., Raveendran R., Salameh S., Friedman-Ezra A., Yavin E., Gibson D. (2015). On the Stability of Pt IV Pro-Drugs with Haloacetato Ligands in the Axial Positions. Chem. A Eur. J..

[35] Ambrus A., Chen D., Dai J., Jones R.A., Yang D. (2005). Solution Structure of the Biologically Relevant G-Quadruplex Element in the Human c-MYC Promoter. Implications for G-Quadruplex Stabilization. Biochemistry.

[36] Mulliri S., Laaksonen A., Spanu P., Farris R., Farci M., Mingoia F., Roviello G.N., Mocci F. (2021). Spectroscopic and In Silico Studies on the Interaction of Substituted Pyrazolo [1,2-a]Benzo [1–4]Tetrazine-3-One Derivatives with c-Myc G4-DNA. Int. J. Mol. Sci..

[37] Bonsignore R., Terenzi A., Spinello A., Martorana A., Lauria A., Almerico A.M., Keppler B.K., Barone G. (2016). G-Quadruplex vs. Duplex-DNA Binding of Nickel(II) and Zinc(II) Schiff Base Complexes. J. Inorg. Biochem..

[38] Bonsignore R., Russo F., Terenzi A., Spinello A., Lauria A., Gennaro G., Almerico A.M., Keppler B.K., Barone G. (2018). The Interaction of Schiff Base Complexes of Nickel(II) and Zinc(II) with Duplex and G-Quadruplex DNA. J. Inorg. Biochem..

[39] Kollman P.A., Massova I., Reyes C., Kuhn B., Huo S., Chong L., Lee M., Lee T., Duan Y., Wang W. (2000). Calculating Structures and Free Energies of Complex Molecules: Combining Molecular Mechanics and Continuum Models. Acc. Chem. Res..

[40] Shan Y., Kim E.T., Eastwood M.P., Dror R.O., Seeliger M.A., Shaw D.E. (2011). How Does a Drug Molecule Find Its Target Binding Site?. J. Am. Chem. Soc..

[41] Decherchi S., Berteotti A., Bottegoni G., Rocchia W., Cavalli A. (2015). The Ligand Binding Mechanism to Purine Nucleoside Phosphorylase Elucidated via Molecular Dynamics and Machine Learning. Nat. Commun..

[42] O’Hagan M.P., Haldar S., Morales J.C., Mulholland A.J., Galan M.C. (2021). Enhanced Sampling Molecular Dynamics Simulations Correctly Predict the Diverse Activities of a Series of Stiff-Stilbene G-Quadruplex DNA Ligands. Chem. Sci..

[43] Castelli M., Serapian S.A., Marchetti F., Triveri A., Pirota V., Torielli L., Collina S., Doria F., Freccero M., Colombo G. (2021). New Perspectives in Cancer Drug Development: Computational Advances with an Eye to Design. RSC Med. Chem..

[44] Spinello A., Barone G., Grunenberg J. (2016). Molecular Recognition of Naphthalene Diimide Ligands by Telomeric Quadruplex-DNA: The Importance of the Protonation State and Mediated Hydrogen Bonds. Phys. Chem. Chem. Phys..

[45] Frisch M.J., Trucks G.W., Schlegel H.B., Scuseria G.E., Robb M.A., Cheeseman J.R., Scalmani G., Barone V., Petersson G.A., Nakatsuji H. (2016). G16_C01, Gaussian 16, Revision C.01.

[46] Becke A.D. (1993). Density-functional Thermochemistry. III. The Role of Exact Exchange. J. Chem. Phys..

[47] Lee C., Yang W., Parr R.G. (1988). Development of the Colle-Salvetti Correlation-Energy Formula into a Functional of the Electron Density. Phys. Rev. B.

[48] Grimme S., Antony J., Ehrlich S., Krieg H. (2010). A Consistent and Accurate Ab Initio Parametrization of Density Functional Dispersion Correction (DFT-D) for the 94 Elements H-Pu. J. Chem. Phys..

[49] Andrae D., Häussermann U., Dolg M., Stoll H., Preuss H. (1990). Energy-Adjustedab Initio Pseudopotentials for the Second and Third Row Transition Elements. Theor. Chim. Acta.

[50] Miertuš S., Tomasi J. (1982). Approximate Evaluations of the Electrostatic Free Energy and Internal Energy Changes in Solution Processes. Chem. Phys..

[51] Miertuš S., Scrocco E., Tomasi J. (1981). Electrostatic Interaction of a Solute with a Continuum. A Direct Utilizaion of AB Initio Molecular Potentials for the Prevision of Solvent Effects. Chem. Phys..

[52] Fukui K. (1970). A Formulation of the Reaction Coordinate. J. Phys. Chem..

[53] Shelley J.C., Cholleti A., Frye L.L., Greenwood J.R., Timlin M.R., Uchimaya M. (2007). Epik: A Software Program for PK a Prediction and Protonation State Generation for Drug-like Molecules. J. Comput. Aided Mol. Des..

[54] Li P., Merz K.M. (2016). MCPB.Py: A Python Based Metal Center Parameter Builder. J. Chem. Inf. Model..

[55] Case D.A., Betz R.M., Cerutti D.S., Cheatham T.E., Darden T.A., Duke R.E., Giese T.J., Gohlke H., Goetz A.W., Homeyer N. (2016). AMBER 2016.

[56] Ivani I., Dans P.D., Noy A., Pérez A., Faustino I., Hospital A., Walther J., Andrio P., Goñi R., Balaceanu A. (2016). Parmbsc1: A Refined Force Field for DNA Simulations. Nat. Methods.

[57] Wang J., Wolf R.M., Caldwell J.W., Kollman P.A., Case D.A. (2004). Development and Testing of a General Amber Force Field. J. Comput. Chem..

[58] Jorgensen W.L., Chandrasekhar J., Madura J.D., Impey R.W., Klein M.L. (1983). Comparison of Simple Potential Functions for Simulating Liquid Water. J. Chem. Phys..

[59] Mahoney M.W., Jorgensen W.L. (2001). Diffusion Constant of the TIP5P Model of Liquid Water. J. Chem. Phys..

[60] Mahoney M.W., Jorgensen W.L. (2000). A Five-Site Model for Liquid Water and the Reproduction of the Density Anomaly by Rigid, Nonpolarizable Potential Functions. J. Chem. Phys..

[61] Miller B.R., McGee T.D., Swails J.M., Homeyer N., Gohlke H., Roitberg A.E. (2012). MMPBSA.Py: An Efficient Program for End-State Free Energy Calculations. J. Chem. Theory Comput..

[62] Roe D.R., Cheatham T.E. (2013). PTRAJ and CPPTRAJ: Software for Processing and Analysis of Molecular Dynamics Trajectory Data. J. Chem. Theory Comput..

[63] Humphrey W., Dalke A., Schulten K. (1996). VMD: Visual Molecular Dynamics. J. Mol. Graph..

