# Supplementary material for: Anticancer Activity, Reduction Mechanism and G-Quadruplex DNA Binding of a Redox-Activated Platinum(IV)–Salphen Complex
Source: Int J Mol Sci. 2022 Dec 8;23(24):15579. doi: 10.3390/ijms232415579 (PMC9778786; doi:10.3390/ijms232415579)
Supplement: Supplementary file 1 [file ijms-23-15579-s001.zip › ijms-2052347-supplementary_rev.pdf]

Supplementary Materials

Anticancer Activity, Reduction mechanism and G-Quadruplex DNA binding of a Redox-Activated Platinum(IV)-Salphen Complex

Vincenzo Vigna [1], Stefano Scoditti [1,*], Angelo Spinello [2], Gloria Mazzone [1] and Emilia Sicilia [1,*]

[1] Department of Chemistry and Chemical Technologies, Università della Calabria, 87036 Arcavacata di Rende, Italy
[2] Dipartimento di Scienze e Tecnologie Biologiche, Chimiche e Farmaceutiche, Viale delle Scienze, Edificio 17, 90128 Palermo, Italy
* Correspondence: stefano.scoditti@unical.it (S.S.); emilia.sicilia@unical.it (E.S.)

Table of Contents

- **Figure S1**. Free energy pathways describing the reduction considering the complete structure of the complex **Pt(IV)-Sal** complex following β-carbon attack mechanisms in presence of $AscH^-$. Relative energies are in kcal mol^{-1} and calculated with respect to the sum of the energies of separated reactants ··· S2
- **Figure S2**. Improved view of π-π interactions of **Pt(II)-SalH$_2^{2+}$** and **Pt(II)-Sal** with the G-Q ··· S3
- **Figure S3**. RMSD plot of MD1 (**A**) and MD2 (**B**) ··· S4
- **Figure S4**. Structural comparison between the binding poses of the complexes in MD1 (Q-G ochre; **Pt(II)-SalH$_2^{2+}$** silver) and MD1_rep (Q-G cyan; **Pt(II)-SalH$_2^{2+}$** blue) ··· S5
- **Figure S5**. Figure S5. Starting point of the targeting 1.5 μs MD run, MD1_targeting··· S6
- **Figure S6**. Structural comparison between the binding poses of **Pt(II)-SalH$_2^{2+}$** in MD1 (Q-G silver; **Pt(II)-SalH$_2^{2+}$** ochre) and MD1_targeting (Q-G orange; **Pt(II)-SalH$_2^{2+}$** green) ··· S6
- **Table S1**. H-bond with a value higher than 10% within MD1 ··· S7
- **Table S2**. Contributions to the MM-GBSA binding free energy for the Q-G-complex adducts. Van der Waals, electrostatic, polar and non-polar contributions to the solvation free energy, total gas phase and solvation binding energy, resulting MM-GBSA binding energy and the estimation of the entropy term by quasi-harmonic analysis are reported ···S7
- Developed parameters for **Pt(II)-SalH$_2^{2+}$** ··· S8
- Developed parameter for **Pt(II)-Sal** ··· S13
- Cartesian coordinates ··· S18

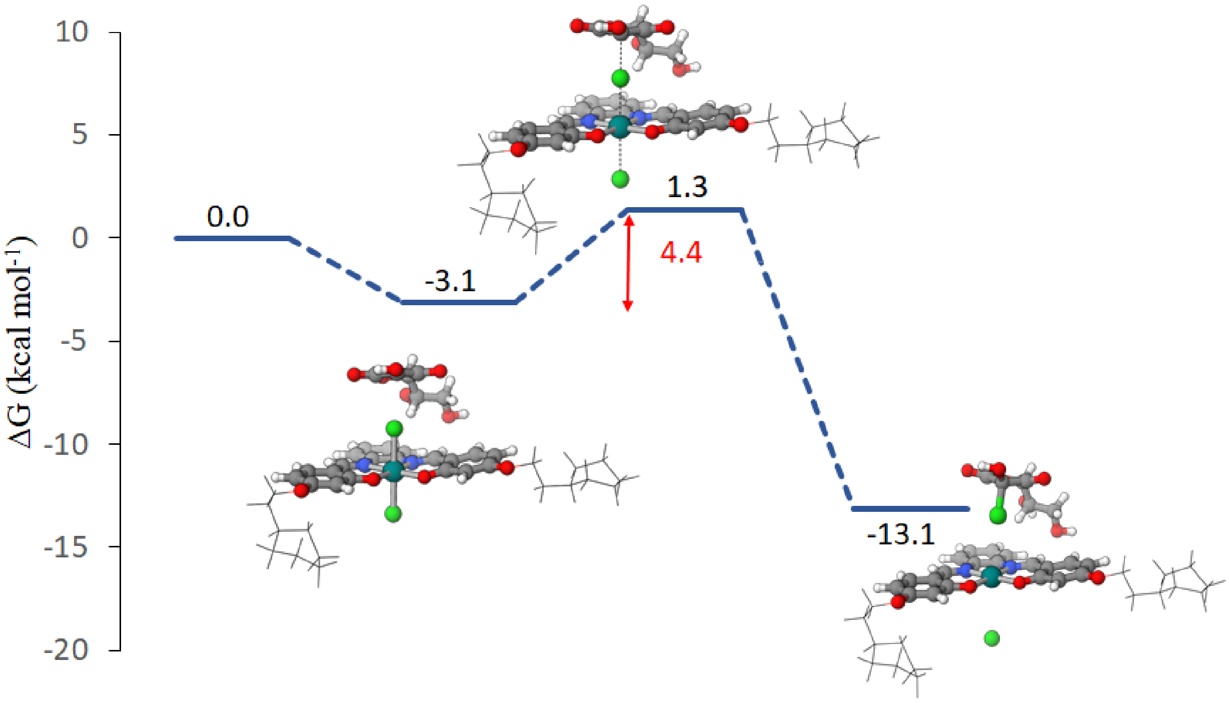

Figure S1. Free energy pathways describing the reduction considering the complete structure of the complex **Pt(IV)-Sal** complex following β-carbon attack mechanisms in presence of AscH^{-}. Relative energies are in kcal mol^{-1} and calculated with respect to the sum of the energies of separated reactants

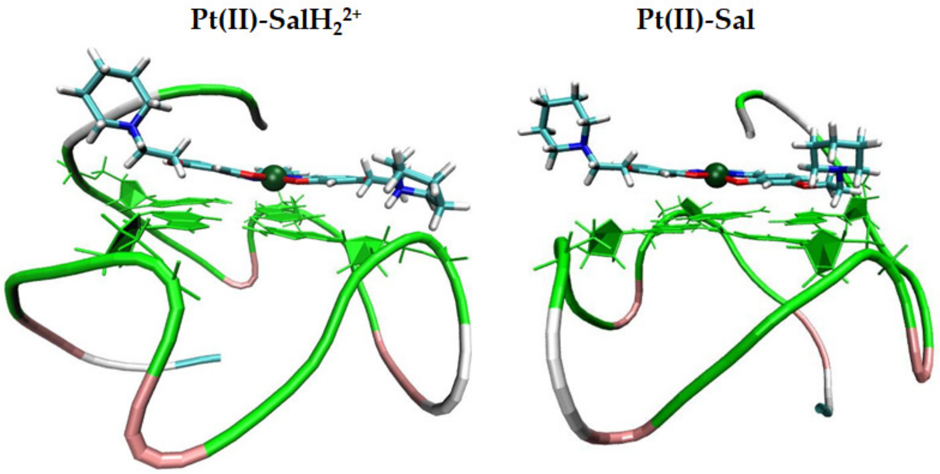

Figure S2. Improved view of π-π interactions of **Pt(II)-SalH$_2$$^{2+}$** and **Pt(II)-Sal** with the G-Q)

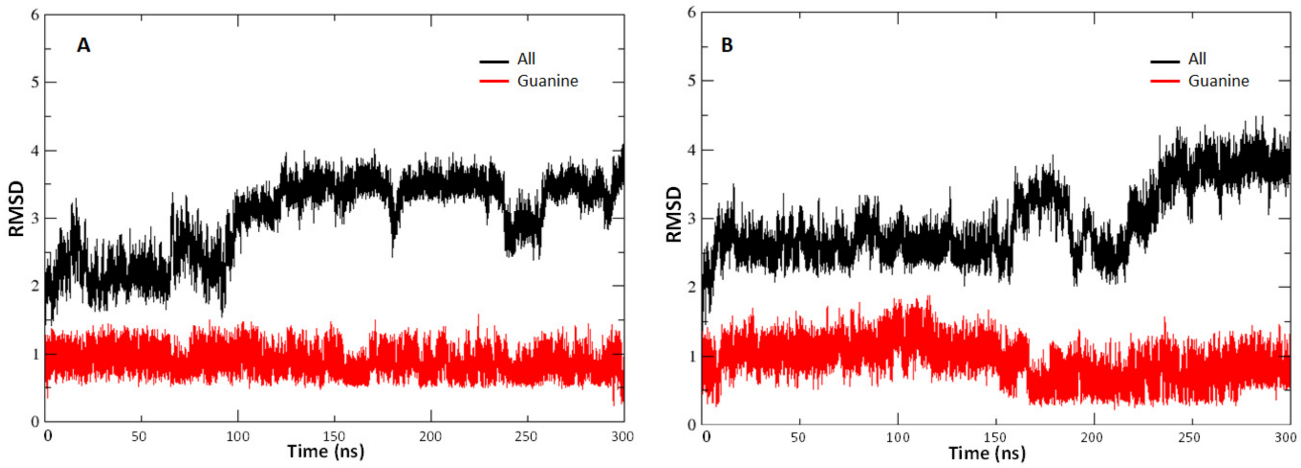

Figure S3. RMSD plot of MD1 (**A**) and MD2(**B**).

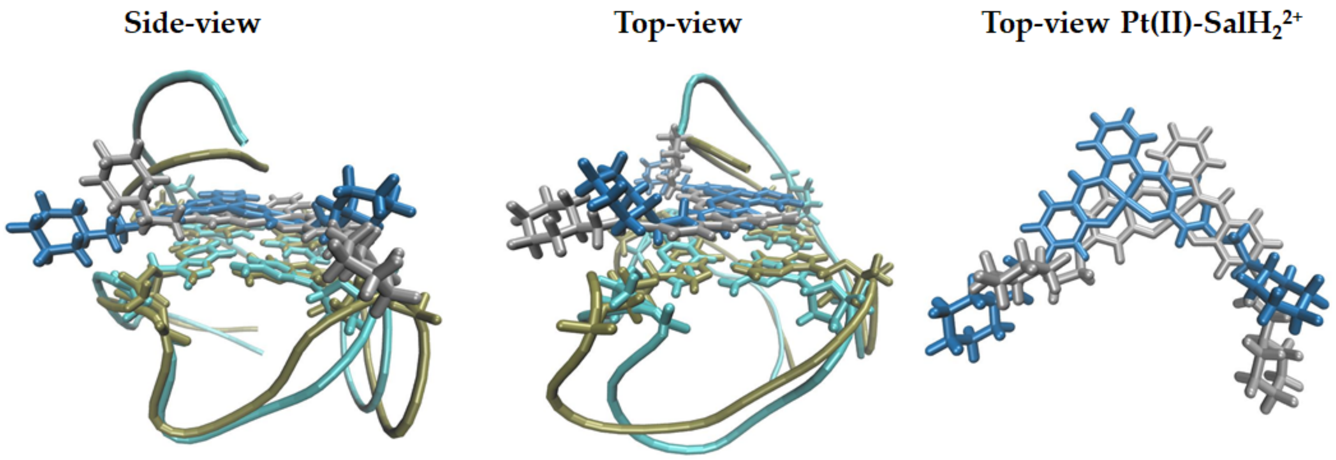

Figure S4. Structural comparison between the binding poses of the **Pt(II)-SalH$_2^{2+}$** in MD1 (Q-G ochre; **Pt(II)-SalH$_2^{2+}$** silver) and MD1_rep (Q-G cyan; **Pt(II)-SalH$_2^{2+}$** blue)

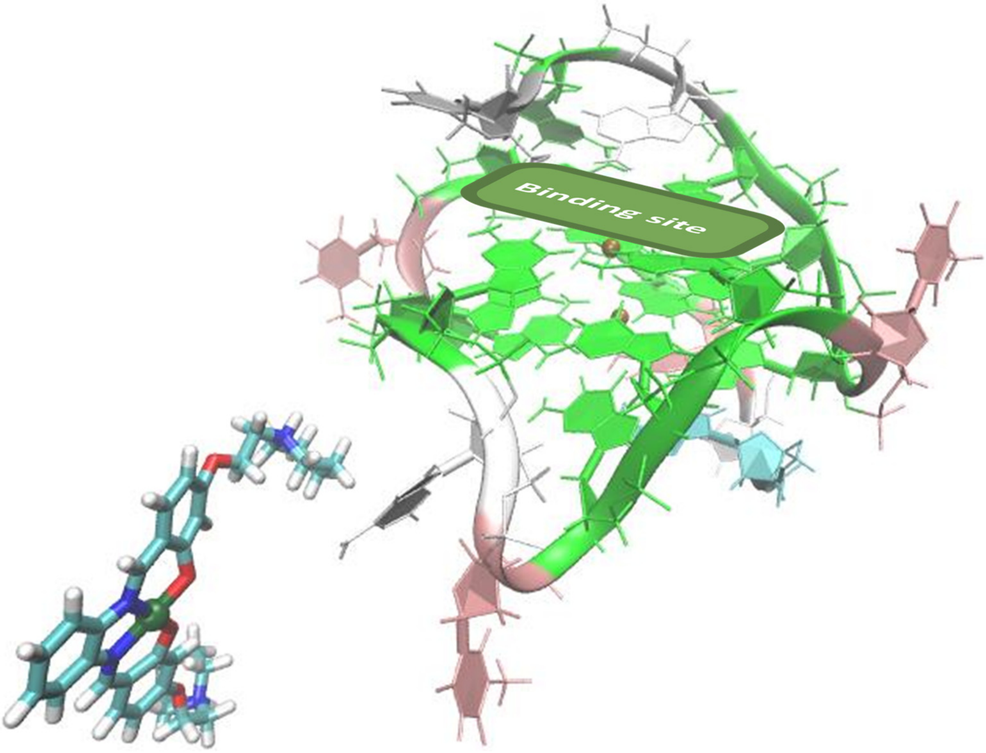

Figure S5. Starting point of the targeting 1.3 μs MD run, MD1_targeting.

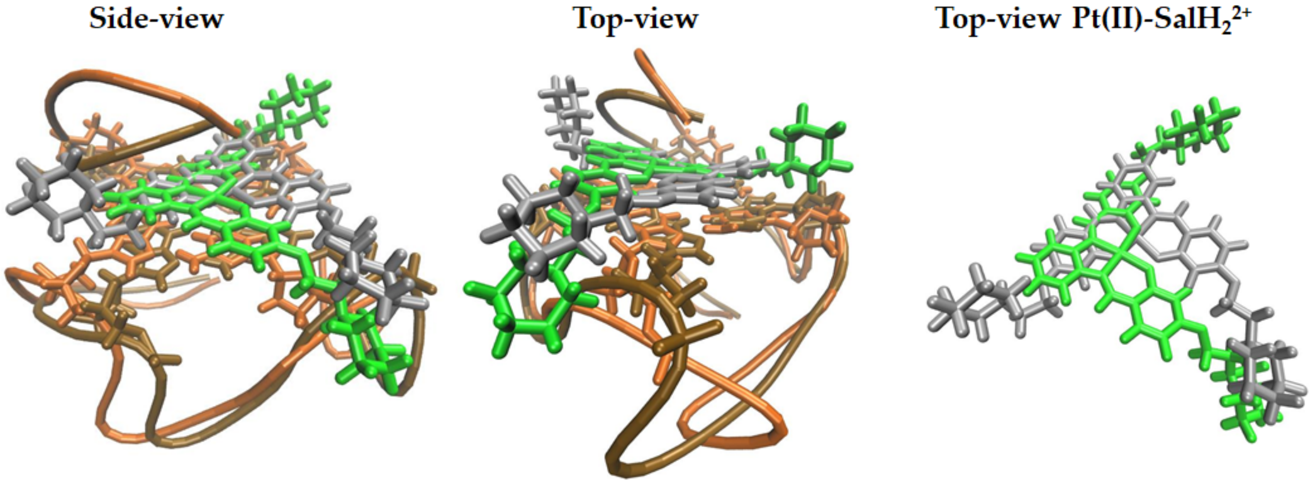

Figure S6. Structural comparison between the binding poses of **Pt(II)-SalH$_2^{2+}$** in MD1 (Q-G silver; **Pt(II)-SalH$_2^{2+}$** ochre) and MD1_targeting (Q-G orange; **Pt(II)-SalH$_2^{2+}$** green).

Table S1. H-bond showing a persistence higher than 10% within MD1.

Acceptor	Donor	Occupancy	Average distance (Å)
DG_14@OP1	AN1_26@N12	51.9%	2.815
DA_3@OP2	AN1_26@N13	47.0%	2.814

Table S2. Contributions to the MM-GBSA binding free energy for the G-quadruplex complex adducts. Van der Waals (VDWAALS), electrostatic (EEL), polar (EGB) and non-polar (ESURF) contributions to the solvation free energy, total gas phase (ΔG gas) and solvation (ΔG solv) binding energy, resulting MM-GBSA binding energy (ΔG TOTAL) and the estimation of the entropy term by quasi-harmonic analysis are reported. All the values are in kcal mol^{-1}.

	MD1	MD2	MD1_rep	MD1_targeting
Energy component				
VDWAALS	-54.9	-45.8	-48.3	-63.3
EEL	-1030.6	-11.4	-981.4	-1022.3
EGB	1024.9	15.8	977.3	1022.1
ESURF	-5.1	-4.1	-4.2	-5.8
ΔG gas	-1047.5	-21.9	-991.5	-1047.3
ΔG solv	1019.8	11.7	973.1	1016.3
ΔG TOTAL	-27.7	-10.2	-18.4	-31.0
Quasi-harmonic entropy approximation	-15.9	-12.3	-5.3	-13.9
ΔG binding	**-11.8**	**2.1**	**-13.1**	**-17.1**

Developed parameters for Pt(II)-SalH$_2^{2+}$

a) Pt^{2+}

```
    0    0    2

This is a remark line
molecule.res
PT1   INT  0
CORRECT     OMIT DU   BEG
  0.0000
   1  DUMM  DU    M    0  -1  -2     0.000      .0        .0      .00000
   2  DUMM  DU    M    1   0  -1     1.449      .0        .0      .00000
   3  DUMM  DU    M    2   1   0     1.523   111.21       .0      .00000
   4  PT    M1    M    3   2   1     1.540   111.208  -180.000  0.247808


LOOP

IMPROPER

DONE
STOP
```

b) SalH$_2^{2+}$

```
    0    0    2

This is a remark line
molecule.res
AN1   INT  0
CORRECT     OMIT DU   BEG
  0.0000
   1  DUMM  DU    M    0  -1  -2     0.000      .0        .0      .00000
   2  DUMM  DU    M    1   0  -1     1.449      .0        .0      .00000
   3  DUMM  DU    M    2   1   0     1.523   111.21       .0      .00000
   4  C36   CT    M    3   2   1     1.540   111.208  -180.000 -0.018710
   5  C35   CT    3    4   3   2     1.531   138.107    53.310 -0.011753
   6  C33   CT    B    5   4   3     1.531   111.146     9.578 -0.082613
   7  H27   HP    E    6   5   4     1.091   111.876    82.687  0.092959
   8  H31   HP    E    6   5   4     1.085   112.302  -153.602  0.092959
   9  H29   HC    E    5   4   3     1.092   111.181   129.706  0.061886
  10  H32   HC    E    5   4   3     1.094   110.092  -112.373  0.061886
  11  H30   HC    E    4   3   2     1.096    60.665   140.095  0.038239
  12  H33   HC    E    4   3   2     1.090   111.180  -123.080  0.038239
  13  C34   CT    M    4   3   2     1.549    51.262   -23.934 -0.036440
  14  H28   HC    E   13   4   3     1.094   110.363   -13.082  0.066614
  15  H34   HC    E   13   4   3     1.091   110.628  -130.717  0.066614
  16  C32   CT    M   13   4   3     1.535   112.097   109.884 -0.113086
  17  H26   HP    E   16  13   4     1.089   112.751    81.971  0.109026
  18  H35   HP    E   16  13   4     1.090   111.802  -155.245  0.109026
  19  N12   N3    M   16  13   4     1.514   109.875   -35.772 -0.063865
  20  H51   H     E   19  16  13     1.021   105.798   -48.811  0.288653
  21  C31   CT    M   19  16  13     1.521   114.566  -164.723 -0.089747
  22  H24   HP    E   21  19  16     1.090   106.424  -165.890  0.115632
  23  H25   HP    E   21  19  16     1.092   107.148    77.741  0.115632
  24  C30   CT    M   21  19  16     1.520   114.194   -43.021  0.032013
  25  H22   H1    E   24  21  19     1.097   107.148  -175.950  0.093250
  26  H23   H1    E   24  21  19     1.095   110.973    66.736  0.093250
  27  O12   OS    M   24  21  19     1.416   108.290   -55.382 -0.148848
```

28	C28	CA	M	27	24	21	1.386	118.896	-176.009	0.051235
29	C29	CA	B	28	27	24	1.409	122.530	-6.148	-0.123785
30	C27	CA	S	29	28	27	1.376	117.643	-179.525	-0.265147
31	H21	HA	E	30	29	28	1.084	118.562	179.952	0.176211
32	H10	HA	E	29	28	27	1.080	122.594	0.704	0.118334
33	C26	CA	M	28	27	24	1.377	115.631	174.220	-0.207961
34	H20	HA	E	33	28	27	1.082	120.624	-1.394	0.126435
35	C23	CA	M	33	28	27	1.418	121.595	-179.901	0.301566
36	O11	Y2	E	35	33	28	1.293	117.135	178.986	-0.369813
37	C25	CA	M	35	33	28	1.448	117.202	-0.800	0.031808
38	C22	CA	M	37	35	33	1.425	125.361	-179.649	0.022532
39	H18	HA	E	38	37	35	1.086	115.338	179.528	0.142948
40	N11	Y4	M	38	37	35	1.306	127.115	-0.724	-0.162133
41	C13	CA	M	40	38	37	1.426	123.470	179.693	0.089966
42	C20	CA	B	41	40	38	1.398	124.794	-3.733	-0.124119
43	C24	CA	B	42	41	40	1.387	120.257	-179.935	-0.129759
44	C21	CA	B	43	42	41	1.394	120.209	0.300	-0.143444
45	C14	CA	S	44	43	42	1.388	120.160	-0.036	-0.121757
46	H15	HA	E	45	44	43	1.081	118.853	179.214	0.130305
47	H17	HA	E	44	43	42	1.083	120.240	179.591	0.158349
48	H19	HA	E	43	42	41	1.083	119.604	179.890	0.154045
49	H16	HA	E	42	41	40	1.081	120.856	-0.365	0.132035
50	C17	CA	M	41	40	38	1.410	115.662	176.573	0.103553
51	N10	Y3	M	50	41	40	1.426	115.657	0.080	-0.164657
52	C18	CA	M	51	50	41	1.306	123.498	-176.560	0.024853
53	H11	HA	E	52	51	50	1.086	117.581	0.471	0.142449
54	C11	CA	M	52	51	50	1.425	127.178	-179.728	0.019809
55	C16	CA	B	54	52	51	1.448	125.318	0.669	0.310164
56	O10	Y1	E	55	54	52	1.293	125.659	-0.646	-0.373068
57	C10	CA	S	55	54	52	1.418	117.192	179.643	-0.205216
58	H13	HA	E	57	55	54	1.082	117.724	179.364	0.125598
59	C15	CA	M	54	52	51	1.415	116.095	-179.038	-0.252787
60	H12	HA	E	59	54	52	1.084	118.354	-0.019	0.173495
61	C12	CA	M	59	54	52	1.375	123.144	179.917	-0.126745
62	H14	HA	E	61	59	54	1.080	119.833	-179.565	0.120746
63	C19	CA	M	61	59	54	1.409	117.684	0.181	0.038728
64	O13	OS	M	63	61	59	1.385	122.573	179.530	-0.142283
65	C37	CT	M	64	63	61	1.415	118.900	6.467	0.035348
66	H36	H1	E	65	64	63	1.096	111.666	58.146	0.092596
67	H37	H1	E	65	64	63	1.095	111.048	-62.011	0.092596
68	C38	CT	M	65	64	63	1.519	108.307	175.933	-0.095408
69	H38	HP	E	68	65	64	1.092	108.990	175.211	0.116359
70	H39	HP	E	68	65	64	1.090	111.069	-64.914	0.116359
71	N13	N3	M	68	65	64	1.520	114.224	55.438	-0.065195
72	H50	H	E	71	68	65	1.022	105.673	159.066	0.288602
73	C39	CT	M	71	68	65	1.540	112.582	-85.769	-0.084443
74	H40	HP	E	73	71	68	1.085	106.209	36.294	0.094475
75	H45	HP	E	73	71	68	1.091	105.193	-79.841	0.094475
76	C41	CT	M	73	71	68	1.530	111.243	158.802	-0.010063
77	H42	HC	E	76	73	71	1.092	107.840	156.580	0.061294
78	H49	HC	E	76	73	71	1.093	109.865	-87.564	0.061294
79	C43	CT	M	76	73	71	1.532	111.159	34.559	-0.021815
80	H44	HC	E	79	76	73	1.096	109.368	57.697	0.038827
81	H48	HC	E	79	76	73	1.090	110.657	174.021	0.038827
82	C42	CT	M	79	76	73	1.548	110.536	-63.900	-0.036115
83	H43	HC	E	82	79	76	1.092	110.438	149.112	0.066466
84	H47	HC	E	82	79	76	1.092	110.589	-93.211	0.066466
85	C40	CT	M	82	79	76	1.536	112.085	26.081	-0.107880
86	H41	HP	E	85	82	79	1.090	111.747	155.181	0.107911
87	H46	HP	E	85	82	79	1.088	112.781	-81.963	0.107911

```
LOOP
  N12  C33
  C25  C27
  C17  C14
  C19  C10
  C40  N13

IMPROPER
  C29  C26  C28  O12
  C28  C27  C29  H10
  C29  C25  C27  H21
  C23  C28  C26  H20
  C26  C25  C23  O11
  C23  C27  C25  C22
  C25  H18  C22  N11
  C20  C17  C13  N11
  C24  C13  C20  H16
  C21  C20  C24  H19
  C24  C14  C21  H17
  C21  C17  C14  H15
  C13  C14  C17  N10
  C11  H11  C18  N10
  C16  C15  C11  C18
  C11  C10  C16  O10
  C16  C19  C10  H13
  C12  C11  C15  H12
  C19  C15  C12  H14
  C12  C10  C19  O13

DONE
STOP
```

c) frcmod file for Pt(II)-Sal

```
REMARK GOES HERE, THIS FILE IS GENERATED BY MCPB.PY
MASS
M1 195.08                              Pt ion
Y1 16.000        0.465               same as os
Y2 16.000        0.465               same as os
Y3 14.010        0.530               same as nc
Y4 14.010        0.530               same as nc

BOND
M1-Y1  135.7    2.0442      Created by Seminario method using MCPB.py
M1-Y2  135.7    2.0442      Created by Seminario method using MCPB.py
M1-Y3  158.0    1.9936      Created by Seminario method using MCPB.py
M1-Y4  158.0    1.9936      Created by Seminario method using MCPB.py
Y1-CA  376.60   1.370       same as ca-os, penalty score=  0.0
Y2-CA  376.60   1.370       same as ca-os, penalty score=  0.0
Y3-CA  467.70   1.352       same as ca-nc, penalty score=  0.0
Y4-CA  467.70   1.352       same as ca-nc, penalty score=  0.0
OS-CA  376.60   1.370       same as ca-os, penalty score=  0.0

ANGL
M1-Y1-CA   133.92     123.66    Created by Seminario method using MCPB.py
M1-Y2-CA   133.92     123.66    Created by Seminario method using MCPB.py
M1-Y3-CA   152.33     118.20    Created by Seminario method using MCPB.py
M1-Y4-CA   152.33     118.20    Created by Seminario method using MCPB.py
Y2-M1-Y1   148.70      88.04    Created by Seminario method using MCPB.py
```

```
Y3-M1-Y1   165.40      94.49    Created by Seminario method using MCPB.py
Y3-M1-Y2   174.13     177.44    Created by Seminario method using MCPB.py
Y4-M1-Y1   174.12     177.44    Created by Seminario method using MCPB.py
Y4-M1-Y2   165.40      94.48    Created by Seminario method using MCPB.py
Y4-M1-Y3   186.23      82.98    Created by Seminario method using MCPB.py
CA-Y3-CA   69.910     109.950   same as ca-nc-ca, penalty score=  0.0
CA-Y4-CA   69.910     109.950   same as ca-nc-ca, penalty score=  0.0
Y1-CA-CA   69.580     119.200   same as ca-ca-os, penalty score=  0.0
Y2-CA-CA   69.580     119.200   same as ca-ca-os, penalty score=  0.0
Y3-CA-CA   69.460     119.720   same as ca-ca-nc, penalty score=  0.0
Y3-CA-HA   51.000     118.360   same as h4-ca-nc, penalty score=  1.3
Y4-CA-CA   69.460     119.720   same as ca-ca-nc, penalty score=  0.0
Y4-CA-HA   51.000     118.360   same as h4-ca-nc, penalty score=  1.3
CA-OS-CT   62.520     117.960   same as c3-os-ca, penalty score=  0.0
CA-CA-OS   69.580     119.200   same as ca-ca-os, penalty score=  0.0

DIHE
CA-Y2-M1-Y1    3       0.00       0.00   3.0    Treat as zero by MCPB.py
CA-Y3-CA-CA    2        9.6      180.0   2.0      same as X -ca-nd-X , penalty score=
0.0
CA-Y3-M1-Y1    3       0.00       0.00   3.0    Treat as zero by MCPB.py
CA-Y3-M1-Y2    3       0.00       0.00   3.0    Treat as zero by MCPB.py
CA-Y4-CA-CA    2        9.6      180.0   2.0      same as X -ca-nd-X , penalty score=
0.0
CA-Y4-M1-Y1    3       0.00       0.00   3.0    Treat as zero by MCPB.py
CA-Y4-M1-Y2    3       0.00       0.00   3.0    Treat as zero by MCPB.py
CA-Y4-M1-Y3    3       0.00       0.00   3.0    Treat as zero by MCPB.py
HA-CA-Y3-CA    2        9.6      180.0   2.0      same as X -ca-nd-X , penalty score=
0.0
HA-CA-Y4-CA    2        9.6      180.0   2.0      same as X -ca-nd-X , penalty score=
0.0
M1-Y1-CA-CA    3       0.00       0.00   3.0    Treat as zero by MCPB.py
M1-Y2-CA-CA    3       0.00       0.00   3.0    Treat as zero by MCPB.py
M1-Y3-CA-CA    3       0.00       0.00   3.0    Treat as zero by MCPB.py
M1-Y3-CA-HA    3       0.00       0.00   3.0    Treat as zero by MCPB.py
M1-Y4-CA-CA    3       0.00       0.00   3.0    Treat as zero by MCPB.py
M1-Y4-CA-HA    3       0.00       0.00   3.0    Treat as zero by MCPB.py
Y1-CA-CA-CA    4       14.5      180.0   2.0    same as X -ca-ca-X , penalty score=  0.0
Y1-CA-CA-HA    4       14.5      180.0   2.0    same as X -ca-ca-X , penalty score=  0.0
Y2-CA-CA-CA    4       14.5      180.0   2.0    same as X -ca-ca-X , penalty score=  0.0
Y2-CA-CA-HA    4       14.5      180.0   2.0    same as X -ca-ca-X , penalty score=  0.0
Y2-M1-Y1-CA    3       0.00       0.00   3.0    Treat as zero by MCPB.py
Y3-CA-CA-CA    4       14.5      180.0   2.0    same as X -ca-ca-X , penalty score=  0.0
Y3-CA-CA-HA    4       14.5      180.0   2.0    same as X -ca-ca-X , penalty score=  0.0
Y3-CA-CA-Y4    4       14.5      180.0   2.0    same as X -ca-ca-X , penalty score=  0.0
Y3-M1-Y1-CA    3       0.00       0.00   3.0    Treat as zero by MCPB.py
Y3-M1-Y2-CA    3       0.00       0.00   3.0    Treat as zero by MCPB.py
Y4-CA-CA-CA    4       14.5      180.0   2.0    same as X -ca-ca-X , penalty score=  0.0
Y4-CA-CA-HA    4       14.5      180.0   2.0    same as X -ca-ca-X , penalty score=  0.0
Y4-M1-Y1-CA    3       0.00       0.00   3.0    Treat as zero by MCPB.py
Y4-M1-Y2-CA    3       0.00       0.00   3.0    Treat as zero by MCPB.py
Y4-M1-Y3-CA    3       0.00       0.00   3.0    Treat as zero by MCPB.py
CA-CA-OS-CT    2       1.800    180.000  2.0    same as X -ca-os-X , penalty score=  0.0

IMPR
CA-CA-CA-Y4         1.1          180.0         2.0          Using the default value
CA-HA-CA-Y3         1.1          180.0         2.0          Same as X -X -ca-ha, penalty
score=  6.0 (use general term))
CA-CA-CA-Y1         1.1          180.0         2.0          Using the default value
CA-CA-CA-Y2         1.1          180.0         2.0          Using the default value
```

```
CA-HA-CA-Y4         1.1          180.0         2.0          Same as X -X -ca-ha, penalty
score=  6.0 (use general term))
CA-CA-CA-Y3         1.1          180.0         2.0          Using the default value

NONB
  M1          1.2660  0.0030764200       CM set for Pt2+ ion in TIP3P water from Li et
al. JCTC, 2013, 9, 2733
  Y1          1.6837  0.1700             same as os
  Y2          1.6837  0.1700             same as os
  Y3          1.8240  0.1700             same as nc
  Y4          1.8240  0.1700             same as nc
```

Developed parameter for Pt(II)-Sal

a) Pt^{2+}

```
    0    0    2

This is a remark line
molecule.res
PT    INT  0
CORRECT     OMIT DU   BEG
  0.0000
   1  DUMM  DU    M    0  -1  -2     0.000      .0        .0      .00000
   2  DUMM  DU    M    1   0  -1     1.449      .0        .0      .00000
   3  DUMM  DU    M    2   1   0     1.523   111.21       .0      .00000
   4  PT    M1    M    3   2   1     1.540   111.208  -180.000  0.196459


LOOP

IMPROPER

DONE
STOP
```

b) Sal

```
    0    0    2

This is a remark line
molecule.res
AN1   INT  0
CORRECT     OMIT DU   BEG
  0.0000
   1  DUMM  DU    M    0  -1  -2     0.000      .0        .0      .00000
   2  DUMM  DU    M    1   0  -1     1.449      .0        .0      .00000
   3  DUMM  DU    M    2   1   0     1.523   111.21       .0      .00000
   4  C36   CT    M    3   2   1     1.540   111.208  -180.000  0.007998
   5  C35   CT    3    4   3   2     1.531   152.317    21.697 -0.024995
   6  C33   CT    B    5   4   3     1.544   110.831    28.490 -0.055119
   7  H27   HC    E    6   5   4     1.095   110.213    92.215  0.063197
   8  H31   HC    E    6   5   4     1.098   109.615  -150.138  0.063197
   9  H29   HC    E    5   4   3     1.093   111.323   150.614  0.023449
  10  H32   HC    E    5   4   3     1.095   108.904   -92.350  0.023449
  11  H30   HC    E    4   3   2     1.094    71.016   116.763  0.006445
  12  H33   HC    E    4   3   2     1.094    94.772  -136.747  0.006445
  13  C34   CT    M    4   3   2     1.543    48.812   -23.717 -0.054060
  14  H28   HC    E   13   4   3     1.094   109.673     4.472  0.022168
  15  H34   HC    E   13   4   3     1.095   110.273  -113.145  0.022168
  16  C32   CT    M   13   4   3     1.546   110.813   125.963 -0.045176
  17  H26   HC    E   16  13   4     1.105   110.060    94.931  0.060354
  18  H35   HC    E   16  13   4     1.094   110.103  -148.187  0.060354
  19  N12   NT    M   16  13   4     1.458   109.545   -28.639 -0.214940
  20  C31   CT    M   19  16  13     1.450   116.066  -153.377 -0.138012
  21  H24   HC    E   20  19  16     1.095   108.522   176.766  0.081490
  22  H25   HC    E   20  19  16     1.098   108.160    60.964  0.081490
  23  C30   CT    M   20  19  16     1.532   117.902   -60.113  0.125693
  24  H22   HC    E   23  20  19     1.098   109.519   168.311  0.042876
  25  H23   HC    E   23  20  19     1.096   110.635    48.841  0.042876
```

26	O12	OS	M	23	20	19	1.432	108.463	-71.875	-0.209437
27	C28	CA	M	26	23	20	1.356	120.112	176.104	0.151568
28	C29	CA	B	27	26	23	1.420	123.382	2.932	-0.103448
29	C27	CA	S	28	27	26	1.369	118.359	179.921	-0.317322
30	H21	HA	E	29	28	27	1.086	118.806	-179.935	0.171795
31	H10	HA	E	28	27	26	1.080	121.447	0.288	0.097223
32	C26	CA	M	27	26	23	1.381	115.981	-177.126	-0.228662
33	H20	HA	E	32	27	26	1.082	119.778	-0.148	0.117501
34	C23	CA	M	32	27	26	1.413	122.194	-179.935	0.293205
35	O11	Y2	E	34	32	27	1.295	117.098	-179.976	-0.348131
36	C25	CA	M	34	32	27	1.452	117.590	-0.095	0.016629
37	C22	CA	M	36	34	32	1.416	125.557	-179.960	0.030818
38	H18	HA	E	37	36	34	1.087	115.299	-179.909	0.125463
39	N11	Y4	M	37	36	34	1.313	127.250	0.123	-0.167464
40	C13	CA	M	39	37	36	1.419	123.870	179.972	0.085100
41	C20	CA	B	40	39	37	1.399	124.989	0.142	-0.121897
42	C24	CA	B	41	40	39	1.388	120.591	179.969	-0.161977
43	C21	CA	B	42	41	40	1.395	120.052	0.004	-0.160685
44	C14	CA	S	43	42	41	1.388	120.131	-0.013	-0.127872
45	H15	HA	E	44	43	42	1.082	118.950	-179.925	0.123460
46	H17	HA	E	43	42	41	1.084	120.274	-179.975	0.141545
47	H19	HA	E	42	41	40	1.083	119.641	-179.985	0.140752
48	H16	HA	E	41	40	39	1.081	120.475	-0.037	0.123005
49	C17	CA	M	40	39	37	1.417	115.693	-179.897	0.100419
50	N10	Y3	M	49	40	39	1.419	115.693	0.000	-0.161942
51	C18	CA	M	50	49	40	1.313	123.918	179.883	0.027650
52	H11	HA	E	51	50	49	1.087	117.446	0.010	0.123463
53	C11	CA	M	51	50	49	1.416	127.292	179.978	0.013118
54	C16	CA	B	53	51	50	1.452	125.522	-0.003	0.269689
55	O10	Y1	E	54	53	51	1.295	125.310	-0.028	-0.341131
56	C10	CA	S	54	53	51	1.412	117.589	179.949	-0.210503
57	H13	HA	E	56	54	53	1.081	118.061	179.901	0.112481
58	C15	CA	M	53	51	50	1.423	116.432	179.992	-0.297168
59	H12	HA	E	58	53	51	1.086	117.986	0.169	0.167918
60	C12	CA	M	58	53	51	1.370	123.207	-179.937	-0.118413
61	H14	HA	E	60	58	53	1.079	120.232	-179.715	0.100698
62	C19	CA	M	60	58	53	1.421	118.325	0.028	0.155854
63	O13	OS	M	62	60	58	1.356	123.383	-179.913	-0.213952
64	C37	CT	M	63	62	60	1.432	120.093	-3.085	0.131861
65	H36	HC	E	64	63	62	1.098	109.789	64.330	0.042004
66	H37	HC	E	64	63	62	1.096	110.012	-54.896	0.042004
67	C38	CT	M	64	63	62	1.531	108.462	-176.007	-0.150056
68	H38	HC	E	67	64	63	1.098	106.727	-166.268	0.083322
69	H39	HC	E	67	64	63	1.095	108.049	-51.593	0.083322
70	N13	NT	M	67	64	63	1.449	117.953	71.864	-0.203133
71	C39	CT	M	70	67	64	1.471	115.766	-75.506	-0.056541
72	H40	HC	E	71	70	67	1.098	112.303	53.575	0.063270
73	H45	HC	E	71	70	67	1.097	107.220	-63.809	0.063270
74	C41	CT	M	71	70	67	1.544	110.346	176.222	-0.026480
75	H42	HC	E	74	71	70	1.093	109.501	149.353	0.024357
76	H49	HC	E	74	71	70	1.096	109.725	-94.211	0.024357
77	C43	CT	M	74	71	70	1.531	110.809	26.076	0.010459
78	H44	HC	E	77	74	71	1.094	109.095	56.903	0.004603
79	H48	HC	E	77	74	71	1.094	110.886	174.612	0.004603
80	C42	CT	M	77	74	71	1.544	109.782	-63.188	-0.049807
81	H43	HC	E	80	77	74	1.094	109.644	154.845	0.021791
82	H47	HC	E	80	77	74	1.095	110.262	-87.494	0.021791
83	C40	CT	M	80	77	74	1.546	110.724	33.413	-0.054199
84	H41	HC	E	83	80	77	1.094	110.049	148.287	0.061023
85	H46	HC	E	83	80	77	1.106	110.063	-94.930	0.061023

```
LOOP
  N12  C33
  C25  C27
  C17  C14
  C19  C10
  C40  N13

IMPROPER
  C29  C26  C28  O12
  C28  C27  C29  H10
  C29  C25  C27  H21
  C23  C28  C26  H20
  C26  C25  C23  O11
  C23  C27  C25  C22
  C25  N11  C22  H18
  C20  C17  C13  N11
  C13  C24  C20  H16
  C20  C21  C24  H19
  C24  C14  C21  H17
  C21  C17  C14  H15
  C13  C14  C17  N10
  C11  N10  C18  H11
  C16  C15  C11  C18
  C11  C10  C16  O10
  C16  C19  C10  H13
  C11  C12  C15  H12
  C15  C19  C12  H14
  C10  C12  C19  O13

DONE
STOP
```

c) frcmod file for Pt(II)-Sal

```
REMARK GOES HERE, THIS FILE IS GENERATED BY MCPB.PY
MASS
M1 195.08                 Pt ion
Y1 16.000     0.465       same as os
Y2 16.000     0.465       same as os
Y3 14.010     0.530       same as nc
Y4 14.010     0.530       same as nc

BOND
M1-Y1  141.8   2.0362    Created by Seminario method using MCPB.py
M1-Y2  141.8   2.0362    Created by Seminario method using MCPB.py
M1-Y3  157.5   1.9940    Created by Seminario method using MCPB.py
M1-Y4  157.5   1.9940    Created by Seminario method using MCPB.py
Y1-CA  376.60  1.370     same as ca-os, penalty score=  0.0
Y2-CA  376.60  1.370     same as ca-os, penalty score=  0.0
Y3-CA  467.70  1.352     same as ca-nc, penalty score=  0.0
Y4-CA  467.70  1.352     same as ca-nc, penalty score=  0.0
OS-CA  376.60  1.370     same as ca-os, penalty score=  0.0

ANGL
M1-Y1-CA  132.94   123.90   Created by Seminario method using MCPB.py
M1-Y2-CA  132.94   123.90   Created by Seminario method using MCPB.py
M1-Y3-CA  154.63   118.02   Created by Seminario method using MCPB.py
M1-Y4-CA  154.63   118.02   Created by Seminario method using MCPB.py
```

Y2-M1-Y1 152.06 87.62 Created by Seminario method using MCPB.py
Y3-M1-Y1 165.47 94.68 Created by Seminario method using MCPB.py
Y3-M1-Y2 165.81 177.70 Created by Seminario method using MCPB.py
Y4-M1-Y1 165.80 177.70 Created by Seminario method using MCPB.py
Y4-M1-Y2 165.47 94.68 Created by Seminario method using MCPB.py
Y4-M1-Y3 181.46 83.01 Created by Seminario method using MCPB.py
CA-Y3-CA 69.910 109.950 same as ca-nc-ca, penalty score= 0.0
CA-Y4-CA 69.910 109.950 same as ca-nc-ca, penalty score= 0.0
Y1-CA-CA 69.580 119.200 same as ca-ca-os, penalty score= 0.0
Y2-CA-CA 69.580 119.200 same as ca-ca-os, penalty score= 0.0
Y3-CA-CA 69.460 119.720 same as ca-ca-nc, penalty score= 0.0
Y3-CA-HA 51.000 118.360 same as h4-ca-nc, penalty score= 1.3
Y4-CA-CA 69.460 119.720 same as ca-ca-nc, penalty score= 0.0
Y4-CA-HA 51.000 118.360 same as h4-ca-nc, penalty score= 1.3
HC-CT-NT 49.550 109.800 same as hc-c3-n3, penalty score= 0.0
CA-CA-OS 69.580 119.200 same as ca-ca-os, penalty score= 0.0
HC-CT-OS 51.050 108.700 same as hc-c3-os, penalty score= 0.0
CA-OS-CT 62.520 117.960 same as c3-os-ca, penalty score= 0.0

DIHE
CA-Y2-M1-Y1 3 0.00 0.00 3.0 Treat as zero by MCPB.py
CA-Y3-CA-CA 2 9.6 180.0 2.0 same as X -ca-nd-X , penalty score= 0.0
CA-Y3-M1-Y1 3 0.00 0.00 3.0 Treat as zero by MCPB.py
CA-Y3-M1-Y2 3 0.00 0.00 3.0 Treat as zero by MCPB.py
CA-Y4-CA-CA 2 9.6 180.0 2.0 same as X -ca-nd-X , penalty score= 0.0
CA-Y4-M1-Y1 3 0.00 0.00 3.0 Treat as zero by MCPB.py
CA-Y4-M1-Y2 3 0.00 0.00 3.0 Treat as zero by MCPB.py
CA-Y4-M1-Y3 3 0.00 0.00 3.0 Treat as zero by MCPB.py
HA-CA-Y3-CA 2 9.6 180.0 2.0 same as X -ca-nd-X , penalty score= 0.0
HA-CA-Y4-CA 2 9.6 180.0 2.0 same as X -ca-nd-X , penalty score= 0.0
M1-Y1-CA-CA 3 0.00 0.00 3.0 Treat as zero by MCPB.py
M1-Y2-CA-CA 3 0.00 0.00 3.0 Treat as zero by MCPB.py
M1-Y3-CA-CA 3 0.00 0.00 3.0 Treat as zero by MCPB.py
M1-Y3-CA-HA 3 0.00 0.00 3.0 Treat as zero by MCPB.py
M1-Y4-CA-CA 3 0.00 0.00 3.0 Treat as zero by MCPB.py
M1-Y4-CA-HA 3 0.00 0.00 3.0 Treat as zero by MCPB.py
Y1-CA-CA-CA 4 14.5 180.0 2.0 same as X -ca-ca-X , penalty score= 0.0
Y1-CA-CA-HA 4 14.5 180.0 2.0 same as X -ca-ca-X , penalty score= 0.0
Y2-CA-CA-CA 4 14.5 180.0 2.0 same as X -ca-ca-X , penalty score= 0.0
Y2-CA-CA-HA 4 14.5 180.0 2.0 same as X -ca-ca-X , penalty score= 0.0
Y2-M1-Y1-CA 3 0.00 0.00 3.0 Treat as zero by MCPB.py
Y3-CA-CA-CA 4 14.5 180.0 2.0 same as X -ca-ca-X , penalty score= 0.0
Y3-CA-CA-HA 4 14.5 180.0 2.0 same as X -ca-ca-X , penalty score= 0.0
Y3-CA-CA-Y4 4 14.5 180.0 2.0 same as X -ca-ca-X , penalty score= 0.0
Y3-M1-Y1-CA 3 0.00 0.00 3.0 Treat as zero by MCPB.py
Y3-M1-Y2-CA 3 0.00 0.00 3.0 Treat as zero by MCPB.py
Y4-CA-CA-CA 4 14.5 180.0 2.0 same as X -ca-ca-X , penalty score= 0.0
Y4-CA-CA-HA 4 14.5 180.0 2.0 same as X -ca-ca-X , penalty score= 0.0
Y4-M1-Y1-CA 3 0.00 0.00 3.0 Treat as zero by MCPB.py
Y4-M1-Y2-CA 3 0.00 0.00 3.0 Treat as zero by MCPB.py
Y4-M1-Y3-CA 3 0.00 0.00 3.0 Treat as zero by MCPB.py
CA-CA-OS-CT 2 1.800 180.000 2.000 same as X -ca-os-X , penalty score= 0.0

IMPR
CA-CA-CA-Y4 1.1 180.0 2.0 Using the default value
CA-HA-CA-Y3 1.1 180.0 2.0 Same as X -X -ca-ha, penalty score= 6.0 (use general term))
CA-CA-CA-Y1 1.1 180.0 2.0 Using the default value
CA-CA-CA-Y2 1.1 180.0 2.0 Using the default value

CA-HA-CA-Y4	1.1	180.0	2.0	Same as X -X -ca-ha, penalty score= 6.0 (use general term))
CA-CA-CA-Y3	1.1	180.0	2.0	Using the default value
CA-CA-CA-HA	1.1	180.0	2.0	Same as X -X -ca-ha, penalty score= 6.0 (use general term))
CA-CA-CA-CA	1.1	180.0	2.0	Using the default value
CA-CA-CA-NC	1.1	180.0	2.0	Using the default value
CA-CA-CA-OS	1.1	180.0	2.0	Using the default value
CA-HA-CA-NC	1.1	180.0	2.0	Same as X -X -ca-ha, penalty score= 6.0 (use general term))

NONB

M1	1.2660	0.0030764200	CM set for Pt2+ ion in TIP3P water from Li et al. JCTC, 2013, 9, 2733
Y1	1.6837	0.1700	same as os
Y2	1.6837	0.1700	same as os
Y3	1.8240	0.1700	same as nc
Y4	1.8240	0.1700	same as nc

Cartesian coordinates

Pt-Salph+Hasc_betaC_ADD

Atom	*X*	*Y*	*Z*
Pt	-0.82801700	0.93062400	0.45139300
Cl	-1.67572700	2.29171000	2.27499800
Cl	0.04117700	-0.44369700	-1.29283400
O	3.63289300	-1.44440400	-2.96334800
C	2.70002300	-2.18380400	-2.55907100
O	-0.04247000	2.58982000	-0.45456400
O	-2.53235100	1.28383000	-0.62323500
C	1.67360900	3.92117700	-1.24086200
C	2.22571700	2.17054900	0.37224400
C	3.98694500	3.60995500	-0.53856100
C	-0.58707200	-1.26684200	2.27669100
C	1.62777600	-1.05916700	3.25634200
C	3.58538800	2.58110900	0.27061800
C	1.24143100	2.85985300	-0.42657900
C	0.65450200	-0.60153800	2.36375100
C	1.97621100	1.10155100	1.26781400
C	3.00825100	4.29135600	-1.30019400
C	-0.82209300	-2.39510700	3.06814900
C	1.38180300	-2.18271000	4.03274800
C	-2.70545400	-1.22325300	1.09701700
C	-3.56564700	0.47787000	-0.59884900
H	-7.53014500	-0.12884500	-2.27347500
C	0.15970400	-2.85123000	3.93674700
C	-3.67988900	-0.72085400	0.19854300
C	-4.64825500	0.83243800	-1.42367100
C	-4.87965000	-1.48261600	0.09766100
C	-5.79615000	0.05940100	-1.48611500
C	-5.91904600	-1.12177300	-0.71613600
H	-6.81964600	-1.72116300	-0.77390000
H	2.84722400	0.70063400	1.77468700
H	4.32312100	2.04270100	0.85443500
H	0.94317400	4.44700700	-1.84150500
H	5.02880000	3.90040100	-0.60127600
H	2.57982000	-0.55446100	3.33401800
H	-1.76687500	-2.91839800	3.01812300
H	2.14408500	-2.53502700	4.71632200
H	-2.97831000	-2.13464000	1.61943300
H	4.27952700	5.48304200	-2.09183200
H	-0.03339600	-3.72548000	4.54576800
H	-4.57154200	1.73015600	-2.02282400
H	-4.96440600	-2.38405200	0.69391400
O	-6.78206300	0.48016600	-2.30821600
O	3.32883900	5.31804000	-2.11799100
N	0.83376600	0.51119300	1.50278100
N	-1.52942000	-0.72103500	1.36605000
C	1.46282800	-2.49184900	-3.10989600
C	2.77347600	-3.01963600	-1.26639600
O	1.51804700	-3.74072800	-1.18788600
O	-0.34431200	-3.92369400	-2.47593800
C	0.76153100	-3.42494200	-2.29851800
O	0.95979800	-2.03323100	-4.30644700
H	0.08886200	-2.44268500	-4.40528200
C	2.97248700	-2.24678400	0.03187100
H	2.10190500	-1.59829500	0.17386400
C	4.23960700	-1.39693200	0.03324600
H	5.10515100	-2.02405000	-0.20383100
H	4.16589600	-0.59499800	-0.70326400
O	4.36414400	-0.86649500	1.36927900
H	5.28102400	-0.61510300	1.51677000
O	3.03821700	-3.21183400	1.08639900
H	3.35086500	-2.72843100	1.86163100
H	3.57300800	-3.76339500	-1.35498600

Pt-Salph+Hasc_betaC_TS

Atom	*X*	*Y*	*Z*
Pt	-0.80196000	0.88248300	0.39599100
Cl	-1.78162800	2.54545800	2.56851400
Cl	0.21166400	-0.73027000	-1.34226800
O	3.34392500	-1.15555700	-2.99371600
C	2.52241600	-1.92768400	-2.51888900
O	0.06141700	2.47683100	-0.56018300
O	-2.48853400	1.24813300	-0.70827900
C	1.81575300	3.80275800	-1.28081900
C	2.26320700	2.12247600	0.44022000
C	4.06825900	3.56107700	-0.38714500
C	-0.64845100	-1.28518900	2.26353200
C	1.60517800	-1.21028100	3.17334700
C	3.61879200	2.55700200	0.42920200
C	1.33061200	2.76759100	-0.45532500
C	0.62290000	-0.67289400	2.33439600
C	1.96629900	1.06327100	1.33718600
C	3.14329300	4.19302700	-1.25223800
C	-0.91115500	-2.41880200	3.03997600
C	1.33076800	-2.33918600	3.93233200
C	-2.78474300	-1.14079800	1.14568200
C	-3.56540500	0.51262400	-0.63795000
H	-7.57884300	0.03541200	-2.24874300
C	0.07208800	-2.94070900	3.86916400
C	-3.74569500	-0.62542900	0.23575400
C	-4.63426200	0.87222200	-1.48476700
C	-4.99103800	-1.31425200	0.19277700
C	-5.82681600	0.17067000	-1.48859800
C	-6.01578800	-0.94408900	-0.63713900
H	-6.95299300	-1.48783200	-0.64778600
H	2.80595000	0.70210900	1.91924100
H	4.31829200	2.05652900	1.08916200
H	1.12555300	4.29375500	-1.95454200
H	5.10721000	3.86879800	-0.37866400
H	2.59155900	-0.77148100	3.21528800
H	-1.87723500	-2.90282900	3.00192400
H	2.10165200	-2.75164800	4.57137900
H	-3.10581100	-2.00708900	1.71556100
H	4.45891800	5.37550500	-1.98355700
H	-0.14346700	-3.82017100	4.46330300
H	-4.50836800	1.72207100	-2.14302500
H	-5.12603300	-2.16813900	0.84751000
O	-6.79576600	0.59266000	-2.33528800
O	3.51678400	5.19331500	-2.08526100
N	0.82045500	0.45264500	1.49166800
N	-1.57553800	-0.69448000	1.36401800
C	1.16751200	-2.20004800	-2.93867500
C	2.77855500	-2.88263700	-1.34267100
O	1.59771500	-3.73393300	-1.26791400
O	-0.28921500	-4.05044900	-2.47498800
C	0.71305200	-3.41256100	-2.24833900
O	0.67865900	-1.84431400	-4.13628400
H	-0.16230500	-2.31012600	-4.26584100
C	3.03589900	-2.25131300	0.02059800
H	2.12442700	-1.73630700	0.33563000
C	4.19529700	-1.25632000	-0.00494500
H	5.08838300	-1.73776300	-0.41519700
H	3.94552600	-0.38417000	-0.61412200
O	4.41361800	-0.87802300	1.36490700
H	5.32470500	-0.58498300	1.46368300
O	3.32757000	-3.31951800	0.91824800
H	3.67945800	-2.89773900	1.71339400
H	3.62271000	-3.52928000	-1.59783400

Pt-Salph+Hasc_betaC_PROD

Atom	*X*	*Y*	*Z*
Pt	-1.22788700	0.66744500	0.13715900
Cl	-3.24953800	1.83470500	3.48250700
Cl	1.23177400	-0.93716200	-1.69491000
O	4.48616300	-0.26760200	-2.40857600
C	3.88307300	-1.20868400	-1.97563000
O	-0.60859700	2.43368100	-0.69867200
O	-2.91969700	0.79705500	-1.01502500
C	0.87423100	4.15113400	-1.17604200
C	1.53258800	2.46896800	0.47511300
C	3.04659000	4.31421500	-0.08886200
C	-0.76064300	-1.55402700	1.88299500
C	1.37848100	-1.11004700	2.95090100
C	2.75841100	3.17769900	0.62065400
C	0.55436100	2.97631200	-0.46076900
C	0.36852800	-0.72367500	2.06308100
C	1.39908400	1.30064200	1.27134900
C	2.08135700	4.80364500	-1.00125000
C	-0.83948800	-2.76751600	2.57393500
C	1.28514500	-2.31757200	3.63005700
C	-2.86819900	-1.69009300	0.72066300
C	-3.87902600	-0.08733400	-0.99239600
H	-7.76485000	-1.08612000	-2.68239000
C	0.17822300	-3.14696100	3.43972700
C	-3.89204600	-1.28192400	-0.17637400
C	-4.98749500	0.14918900	-1.83483100
C	-5.02093100	-2.14408200	-0.26683700
C	-6.06041400	-0.72147500	-1.88837300
C	-6.08555100	-1.89109900	-1.09153900
H	-6.93122900	-2.56723900	-1.13839700
H	2.24617200	1.08170000	1.91265700
H	3.48981200	2.78636400	1.31905100
H	0.15010200	4.54071200	-1.88008000
H	3.99008200	4.83155100	0.03980900
H	2.24708500	-0.48470500	3.10383500
H	-1.68715500	-3.42551200	2.44056700
H	2.07931300	-2.61174500	4.30508000
H	-3.05733500	-2.62098500	1.24617500
H	3.17073400	6.28534600	-1.53624000
H	0.10624800	-4.09097600	3.96580800
H	-4.98667000	1.03979800	-2.45014000
H	-5.03151000	-3.03856900	0.34643600
O	-7.07809400	-0.41004400	-2.72901100
O	2.29994200	5.92165400	-1.73720600
N	0.39135300	0.46329000	1.28102000
N	-1.74281300	-1.06950600	0.97444400
C	2.63159200	-1.84936100	-2.56209600
C	4.24696600	-2.04646900	-0.75000900
O	3.46063200	-3.26925300	-0.87983900
O	1.97584100	-4.17488000	-2.30846500
C	2.62018300	-3.24055000	-1.92822300
O	2.53769300	-1.80455800	-3.90711800
H	1.92953400	-2.50133200	-4.20151500
C	3.95503000	-1.39238100	0.59871800
H	2.87498100	-1.21559700	0.66618700
C	4.69255100	-0.06488800	0.77084000
H	5.76831700	-0.22136200	0.64937600
H	4.35066700	0.67417500	0.04077100
O	4.39064500	0.36341400	2.10627900
H	5.09778800	0.93451800	2.42274800
O	4.36249100	-2.32029700	1.59096900
H	4.38520900	-1.82627200	2.42133800
H	5.29833000	-2.33455100	-0.80238100

Pt-Salph+Cys_ADD

Atom	*X*	*Y*	*Z*
Pt	-0.84520300	0.20750700	-0.29541500
Cl	-2.05197600	-1.74063600	-0.92358100
Cl	0.37719100	2.15667800	0.32991400
S	4.77330400	1.84915300	0.73668100
H	5.30858700	-0.48054100	1.28744900
H	5.12474100	-0.27660700	-0.45586600
C	4.71873900	0.01779700	0.51206500
H	1.56109700	-0.09316300	1.70164700
H	2.91119200	-0.43964900	2.62395300
N	2.58636500	0.03411400	1.77878200
H	2.85590100	1.03811400	1.82384400
C	3.26774300	-0.50228300	0.56846500
C	3.17381400	-2.01261800	0.60646200
O	2.82327900	-2.65264600	1.57014600
O	3.55869400	-2.54422000	-0.55837700
H	3.51070500	-3.51161700	-0.49386800
H	2.70559200	-0.13591900	-0.28843200
O	0.76685700	-0.46394800	-1.34144300
O	-0.04962000	-0.77056700	1.31537100
N	-1.65667600	1.22705000	-1.82415900
N	-2.46908900	0.86534600	0.69875700
C	2.12836600	-0.70654100	-3.18434300
C	0.11003900	0.65668300	-3.42098400
C	1.62612600	0.35221000	-5.32195100
C	-3.26832300	1.72160600	-0.10513800
C	-3.61134500	2.68792100	-2.30603800
C	0.48977300	0.88372400	-4.77574600
C	0.96930900	-0.16663500	-2.60764400
C	-2.85313700	1.89129400	-1.44363200
C	-1.09073000	1.28043900	-3.00225000
C	2.45594900	-0.45564300	-4.50865800
C	-4.41273000	2.37787200	0.35547600
C	-4.75406700	3.32223800	-1.83970000
C	-2.76835900	0.44951600	1.89995400
C	-0.75602500	-1.02785900	2.41042400
H	-0.66591400	-3.21933800	6.14984300
C	-5.15143900	3.17244600	-0.50950200
C	-2.03096500	-0.44400200	2.72198900
C	-0.16764700	-1.90930400	3.32118900
C	-2.62897400	-0.78528600	3.96702100
C	-0.78911600	-2.21211100	4.52767700
C	-2.03901300	-1.64315500	4.85837700
H	-2.51854900	-1.88639900	5.79875500
H	-1.59473700	1.87672500	-3.75591500
H	-0.15270600	1.50274900	-5.39125900
H	2.77066600	-1.33123800	-2.57794200
H	1.89086200	0.54168900	-6.35504900

H	-3.31614500	2.82262500	-3.33706700
H	-4.72825100	2.28061300	1.38503900
H	-5.33152700	3.94097800	-2.51494300
H	-3.70161800	0.80899300	2.32147300
H	3.72372100	-0.77977600	-5.90535000
H	-6.03638900	3.67784100	-0.14400600
H	0.78553500	-2.35848200	3.07272700
H	-3.58963700	-0.34753700	4.21225300
O	-0.14945800	-3.06815100	5.34805200
O	3.58981900	-1.01446900	-4.97839400

Pt-Salph+Cys_TS

Atom	*X*	*Y*	*Z*
Pt	-1.10131400	-0.01710100	-0.30128300
Cl	-5.21933000	-1.43733400	0.42406200
Cl	0.82521000	1.55116200	0.39463400
S	3.14822700	2.24870100	1.19783800
H	4.82754500	0.57626600	1.16283300
H	4.31980600	0.93101100	-0.49717100
C	3.96288900	0.74383800	0.51385100
H	1.34539800	-1.05771700	1.51574000
H	2.79204200	-1.57768500	2.22087800
N	2.33978100	-0.80649400	1.71668500
H	2.32681200	0.02847300	2.31229600
C	3.09057800	-0.53161300	0.44812500
C	3.94208700	-1.76008200	0.15154000
O	4.09256500	-2.67227900	0.92722000
O	4.48342400	-1.67682100	-1.06099000
H	5.03643400	-2.45905400	-1.22112600
H	2.35146700	-0.43259300	-0.34392800
O	0.32954500	-0.94805500	-1.43217200
O	-0.45807000	-1.19231300	1.26806100
N	-1.75250100	1.15822500	-1.77652900
N	-2.52841800	0.90979500	0.74864000
C	1.74350800	-1.19693600	-3.24627200
C	-0.08366700	0.42414000	-3.41362200
C	1.43548600	0.06638100	-5.30449800
C	-3.22177400	1.88371800	-0.01928400
C	-3.46258300	2.92610800	-2.20102500
C	0.35787200	0.69544000	-4.74007600
C	0.63311500	-0.57145600	-2.65156800
C	-2.82031500	2.00565200	-1.36771000
C	-1.19015100	1.18880000	-2.95998500
C	2.14146400	-0.89011200	-4.53708600
C	-4.24088500	2.70268800	0.47504800
C	-4.48093600	3.72491500	-1.69943900
C	-2.82730500	0.58695800	1.97768300
C	-1.06660800	-1.20802800	2.44434300
H	-1.01945300	-3.08571000	6.36212700
C	-4.86690000	3.61626000	-0.36166800
C	-2.18154900	-0.37411800	2.80679800
C	-0.56386700	-2.10780300	3.39585100
C	-2.71262000	-0.50988700	4.11838500
C	-1.11132100	-2.20048900	4.66819000
C	-2.20444200	-1.38912800	5.04041700
H	-2.63147700	-1.46645800	6.03325900
H	-1.59714700	1.88853100	-3.68274900
H	-0.18326100	1.43534900	-5.31886900
H	2.30035100	-1.92714900	-2.67491600
H	1.74904000	0.29428100	-6.31627300
H	-3.17749200	3.02620000	-3.23906600
H	-4.55131500	2.63549700	1.50838400
H	-4.97177400	4.43532700	-2.35293800
H	-3.66211400	1.10421300	2.43788700
H	3.41642200	-1.24186000	-5.92107300
H	-5.65736100	4.24395700	0.03047400
H	0.25669600	-2.75906100	3.12395100
H	-3.55531200	0.11295000	4.39575800
O	-0.55846800	-3.09675800	5.51417200
O	3.22333900	-1.53839200	-5.02302200

Pt-Salph+Cys_PROD

Pt	-0.94773800	-0.09435900	-0.32420000
Cl	-5.65887800	1.64962000	3.87735100
Cl	1.99574900	2.34777700	-1.32704100
S	3.16417700	2.43028200	0.40963900
H	4.62329200	0.78813700	1.19560800
H	4.48175000	0.64919900	-0.56765700
C	3.91995100	0.77048700	0.35649800
H	1.15867000	-0.77713400	1.58333400
H	2.56372700	-0.66977800	2.52629400
N	2.07964400	-0.26883200	1.71944700
H	1.85766900	0.71213400	1.91347400
C	2.90732100	-0.37400900	0.48517000
C	3.58713800	-1.73549800	0.49809500
O	3.55285700	-2.49075100	1.43703000
O	4.21195500	-1.95958500	-0.65721900
H	4.64914800	-2.82604200	-0.62232400
H	2.21339400	-0.36002000	-0.35754500
O	0.57212200	-0.89285500	-1.44398000
O	-0.34267000	-1.30296300	1.24158600
N	-1.58510400	1.11748700	-1.77047100
N	-2.47937900	0.69032800	0.68811200
C	1.96511500	-1.12030100	-3.28006300
C	0.09968300	0.45925200	-3.42210400
C	1.62046500	0.15727400	-5.32209200
C	-3.17545400	1.68589700	-0.05987100
C	-3.33114100	2.86092600	-2.17963500
C	0.52986100	0.75497500	-4.74581900
C	0.84510300	-0.52220100	-2.67177200
C	-2.70286000	1.90758200	-1.37238500
C	-1.02785300	1.18926600	-2.95319600
C	2.35007800	-0.79170000	-4.56864400
C	-4.25955600	2.42458400	0.42408800
C	-4.40980400	3.58568200	-1.69023500
C	-2.84540500	0.30291000	1.88240100
C	-1.03798600	-1.44194100	2.36603300
H	-1.15512800	-3.55694800	6.15708100
C	-4.87194300	3.36816800	-0.39059300
C	-2.21976500	-0.69444000	2.69130200
C	-0.54770800	-2.37031500	3.29464500
C	-2.83889800	-0.94250000	3.94609600
C	-1.18223500	-2.57751500	4.51291200
C	-2.34821900	-1.85782600	4.84558900
H	-2.84173300	-2.02572400	5.79596700
H	-1.45429000	1.88750700	-3.66619100
H	-0.03051900	1.48840400	-5.31498300
H	2.54405800	-1.83608700	-2.71138700
H	1.92499500	0.40468200	-6.33215900
H	-2.98591800	3.04837200	-3.18694300
H	-4.63237200	2.27210800	1.42976700
H	-4.88735600	4.32317200	-2.32369400
H	-3.72018000	0.77147700	2.32835400
H	3.62400100	-1.10058700	-5.96483800
H	-5.71104500	3.93666100	-0.00826700
H	0.34231800	-2.93656000	3.05061700
H	-3.73073800	-0.37476900	4.19090200
O	-0.63502800	-3.49220200	5.34672200
O	3.44568200	-1.41138400	-5.06878300

Pt-Salph+HAsc_LigandBr_ADD

Atom	*X*	*Y*	*Z*
Pt	-1.06721900	-0.34840900	-0.35702200
Cl	-1.69062800	-1.03724800	-2.53878300
Cl	-0.48782700	0.37087000	1.83849000
O	3.01426400	1.98298700	3.08140200
C	3.52198700	1.63222200	1.96916700
O	-0.03168900	-2.09076600	-0.07546400
O	-2.69741100	-1.31179100	0.41016400
C	1.57448500	-3.65085100	-0.64472300
C	1.89101000	-1.35346300	-1.40806700
C	3.57043400	-3.06012100	-1.91252500
C	-1.10883100	2.42275300	-1.08161400
C	1.18069500	2.95400100	-1.71283500
C	3.12702200	-1.76655700	-1.97614200
C	1.09949700	-2.33370300	-0.70958500
C	0.22111900	2.01656400	-1.32678300
C	1.58429600	0.02373600	-1.54177600
C	2.78061800	-4.01173800	-1.23132200
C	-1.46493000	3.76143600	-1.26298400
C	0.81123100	4.28086700	-1.88548800
C	-3.27445200	1.57581600	-0.37552500
C	-3.87715700	-0.74262000	0.50130700
H	-7.98484300	-1.47128600	1.71568700
C	-0.50805100	4.68220800	-1.66986600
C	-4.18828600	0.61689300	0.12842500
C	-4.90991900	-1.54542100	1.01409300
C	-5.52952700	1.06806300	0.29652200
C	-6.20057300	-1.06129200	1.15797600
C	-6.52220500	0.26761500	0.79265200
H	-7.53373100	0.63728200	0.90958700
H	2.34307800	0.63190500	-2.02525000
H	3.74192200	-1.01192500	-2.45149300
H	0.98635900	-4.39338200	-0.12208000
H	4.51935500	-3.34881500	-2.34801100
H	2.21764300	2.66903500	-1.82731900
H	-2.47615400	4.09567100	-1.07704100
H	1.56254100	5.00697600	-2.16928500
H	-3.68765100	2.56108100	-0.56679700
H	4.00455900	-5.44577800	-1.55518500
H	-0.79110900	5.71945600	-1.79919000
H	-4.68305000	-2.56453000	1.29786900
H	-5.76464100	2.08915400	0.01852600
O	-7.12595300	-1.90803200	1.65692700
O	3.15871200	-5.30235900	-1.11251900
N	0.50111700	0.63716000	-1.14243700
N	-2.00266400	1.41480100	-0.62969400
C	3.61556000	2.31832700	0.77110100
C	4.10872700	0.24519100	1.70920600
O	4.60769100	0.29545300	0.34931400
O	4.39758900	1.67607000	-1.42441100
C	4.21593900	1.49202400	-0.22309100
O	3.20346700	3.60359900	0.50132000
H	2.31853000	3.70950100	0.90918800
C	3.07306000	-0.87218700	1.77040400
H	2.30267000	-0.61307000	1.03456600
C	2.37245700	-1.06637400	3.11131000
H	3.09471800	-1.37491400	3.87043900
H	1.88447300	-0.14555500	3.43208000
O	1.43651300	-2.14996900	2.98181200
H	0.72512600	-1.84389100	2.40140400
O	3.72110600	-2.08650700	1.39405400
H	3.05610700	-2.77948200	1.49732500
H	4.94995300	0.02665700	2.37341500
O	0.94461900	3.41407000	2.07568400
H	1.61304600	2.93636400	2.62558900
H	0.37224900	2.70781800	1.74827400

Pt-Salph+HAsc_LigandBr_TS

Atom	*X*	*Y*	*Z*
Pt	-1.02150000	-0.21560000	-0.65290000
Cl	-4.53580000	1.76840000	-2.61830000
Cl	0.14960000	-0.40840000	1.49480000
O	2.15930000	0.33950000	3.08860000
C	3.10550000	0.52270000	2.27260000
O	0.29950000	-1.51500000	-1.51500000
O	-2.26850000	-1.80450000	-0.37300000
C	2.41760000	-2.24060000	-2.08110000
C	2.05880000	0.16540000	-1.77920000
C	4.27350000	-0.69340000	-2.37700000
C	-1.77610000	2.44810000	0.07160000
C	0.07730000	3.87130000	-0.60600000
C	3.43260000	0.35070000	-2.10730000
C	1.53780000	-1.18480000	-1.78240000
C	-0.50770000	2.60280000	-0.53400000
C	1.34230000	1.33790000	-1.43270000
C	3.75530000	-2.00970000	-2.34870000
C	-2.41820000	3.56500000	0.62020000
C	-0.58020000	4.97000000	-0.07370000
C	-3.48040000	0.81020000	0.56620000
C	-3.41680000	-1.71860000	0.25180000
H	-6.79200000	-4.05290000	1.68070000
C	-1.82460000	4.81610000	0.54350000
C	-4.03300000	-0.48820000	0.69120000
C	-4.10120000	-2.92700000	0.47730000
C	-5.31420000	-0.56430000	1.30680000
C	-5.34510000	-2.95010000	1.08530000
C	-5.96680000	-1.75070000	1.50930000
H	-6.93970000	-1.77900000	1.98520000
H	1.90460000	2.26340000	-1.48910000
H	3.83310000	1.35640000	-2.09140000
H	2.03990000	-3.25350000	-2.05190000
H	5.32440000	-0.52430000	-2.57620000
H	1.04350000	4.00820000	-1.06990000
H	-3.37860000	3.46580000	1.10600000
H	-0.11950000	5.94790000	-0.13510000
H	-4.11270000	1.62020000	0.91220000
H	5.45670000	-2.82160000	-2.68290000
H	-2.33320000	5.67340000	0.96620000
H	-3.64170000	-3.85190000	0.15430000
H	-5.78300000	0.35820000	1.62990000
O	-5.93190000	-4.15530000	1.25490000
O	4.53960000	-3.09150000	-2.54780000
N	0.10460000	1.41360000	-1.00820000
N	-2.29930000	1.13200000	0.10100000
C	3.34850000	1.60140000	1.41750000
C	4.19180000	-0.51030000	1.96500000
O	5.12900000	0.18510000	1.10330000
O	5.18550000	2.11590000	-0.06070000
C	4.61530000	1.39360000	0.72740000
O	2.63180000	2.69460000	1.17200000
H	1.81160000	2.71970000	1.74670000
C	3.61110000	-1.69620000	1.19120000
H	3.00430000	-1.26530000	0.38770000
C	2.70880000	-2.60150000	2.02960000
H	3.33650000	-3.24720000	2.64740000

H	2.04990000	-2.02040000	2.67510000
O	1.96220000	-3.46490000	1.16250000
H	1.29630000	-2.91850000	0.72410000
O	4.68440000	-2.44890000	0.63090000
H	4.27750000	-3.16320000	0.12520000
H	4.73520000	-0.82990000	2.85670000
O	0.56450000	2.57290000	2.80790000
H	0.92360000	1.71600000	3.12320000
H	-0.24440000	2.33670000	2.33770000

Pt-Salph+HAsc_LigandBr_PROD

Atom	*X*	*Y*	*Z*
Pt	-0.81916400	-0.48899500	-0.93110400
Cl	-6.22958900	2.70345300	0.77323300
Cl	0.07213500	-0.23094200	1.34322500
O	1.77213700	0.90754800	3.03103700
C	2.80427400	1.06380900	2.32021000
O	0.71344500	-1.75515400	-1.41867300
O	-1.93666400	-2.14670100	-0.52218600
C	2.94344200	-2.32468000	-1.61161900
C	2.33831400	0.04787200	-1.72800000
C	4.68091600	-0.64606100	-1.90894500
C	-1.88366900	2.15705600	-0.69852500
C	-0.08723300	3.65532300	-1.36505500
C	3.72298100	0.33001100	-1.90858700
C	1.94237400	-1.33702800	-1.58617700
C	-0.56881300	2.35516900	-1.18011500
C	1.48265200	1.17531400	-1.65523000
C	4.28016900	-1.99289900	-1.74243400
C	-2.68001200	3.26387300	-0.37807500
C	-0.89205400	4.74259800	-1.05962000
C	-3.48412800	0.43840000	-0.12009300
C	-3.16021800	-2.09374600	-0.05726300
H	-6.47465900	-4.54070400	1.32258200
C	-2.18325100	4.54637500	-0.56171000
C	-3.92888000	-0.88328800	0.12857800
C	-3.76453700	-3.32328300	0.26227500
C	-5.26452500	-1.00168000	0.61192000
C	-5.06705100	-3.38810200	0.72863700
C	-5.83399200	-2.21105100	0.90775700
H	-6.85238000	-2.27476000	1.27331500
H	1.96802200	2.13773300	-1.77540900
H	4.02864300	1.36463400	-1.99826800
H	2.65525200	-3.35758300	-1.47120700
H	5.73022300	-0.39500500	-2.00083200
H	0.91187900	3.82607100	-1.73927000
H	-3.68365500	3.13247600	0.00772200
H	-0.50971200	5.74523900	-1.20463100
H	-4.21705300	1.22039000	0.07114400
H	6.07563000	-2.65847000	-1.72230600
H	-2.80656200	5.39704300	-0.31532200
H	-3.19366300	-4.23289400	0.12964700
H	-5.83531300	-0.08902700	0.74728800
O	-5.56662000	-4.61307000	1.00354600
O	5.17507400	-3.00266500	-1.67136900
N	0.19519600	1.18023100	-1.40736100
N	-2.29762100	0.80801700	-0.54546500
C	3.07504400	2.02869500	1.34601000
C	3.99949100	0.10831300	2.30985100
O	4.98754300	0.76336600	1.47302200
O	5.05126400	2.50799800	0.04550900
C	4.43712400	1.85092600	0.85722000
O	2.31335900	2.99853500	0.84999200
H	1.42404800	3.02608200	1.31133100
C	3.62674400	-1.22617200	1.66018500
H	3.11122700	-0.97416000	0.72752500
C	2.68446200	-2.08734800	2.50153100
H	3.26542900	-2.58334300	3.28195400
H	1.90271800	-1.48606500	2.96619000
O	2.12822800	-3.12482700	1.68372200
H	1.49519200	-2.70521100	1.08580200
O	4.82470300	-1.94145400	1.36729000
H	4.55308200	-2.75184700	0.91909500
H	4.44043600	-0.02717200	3.29952100
O	0.06465900	2.93106100	2.22633900
H	0.42983300	2.16058600	2.71009400
H	-0.66486100	2.56973900	1.70798200

Pt-Salph+Hasc_betaC_ADD

Atom	*X*	*Y*	*Z*
Pt	0.65230900	-0.36124400	-0.82219300
Cl	0.97818700	0.70053100	-2.98995700
Cl	0.30324500	-1.45979900	1.27026500
O	-2.75698600	-2.92715400	3.45675400
C	-1.65474200	-3.45316700	3.16475800
O	-0.60912300	1.14388500	-0.24805500
O	2.16561900	0.76089800	-0.02900500
C	-2.65155700	2.03119200	0.36438500
C	-2.63488600	-0.14155500	-0.74440800
C	-4.75454300	0.86950300	-0.03275000
C	1.10258500	-2.91537900	-2.04768400
C	-1.08079700	-3.66940700	-2.80374200
C	-4.04881500	-0.15349100	-0.61561900
C	-1.91118000	0.98927500	-0.21859200
C	-0.28934900	-2.71385500	-2.16052500
C	-2.06961100	-1.27344000	-1.38788400
C	-4.03431600	1.98180100	0.45842800
C	1.67459000	-4.08313200	-2.56110500
C	-0.49958900	-4.82462400	-3.30625200
C	3.11993800	-1.90211700	-1.16588900
C	3.40966100	0.35095600	-0.00038000
C	0.87575800	-5.03211300	-3.18329200
C	3.89844900	-0.90218800	-0.52329300
C	4.33846500	1.22655200	0.58822100
C	5.28599600	-1.18326900	-0.40330300
C	5.68401700	0.90814100	0.67711400
C	6.17819800	-0.31731600	0.17825300
H	7.22231100	-0.58297000	0.24884800
H	-2.77617700	-2.03102300	-1.70856900
H	-4.58496400	-1.01881200	-0.98749400
H	-2.12275800	2.88891600	0.75810300
H	-5.82871300	0.80009400	0.04586800
H	-2.14743400	-3.52553200	-2.89605200
H	2.73866000	-4.25827400	-2.48425100
H	-1.12111300	-5.56359600	-3.79612200
H	3.66879800	-2.77721700	-1.49859600
H	1.32910200	-5.93259400	-3.57820500
H	3.98464400	2.17187200	0.97764100
H	5.65228900	-2.12699300	-0.79093400
O	6.46663200	1.85024000	1.26622300
O	-4.60528300	3.05914600	1.06203500
N	-0.80305800	-1.52588500	-1.57841700
N	1.83642000	-1.88585600	-1.39980100
C	-0.37179600	-3.23498700	3.65391700
C	-1.49704400	-4.59631900	2.14345200
O	-0.08377900	-4.92031500	2.12606300
O	1.76768500	-4.22045600	3.24069100
C	0.56233300	-4.11863900	3.04479800
O	-0.01419800	-2.35474700	4.64764000
H	0.94026800	-2.45785800	4.76791500
C	-1.95005100	-4.30359200	0.71833700
H	-1.32517900	-3.49490400	0.32613800
C	-3.41801100	-3.89594200	0.63184500
H	-4.04130500	-4.66059100	1.10648700
H	-3.58334000	-2.93941800	1.13040700
O	-3.72683700	-3.80901700	-0.77513100
H	-4.67764600	-3.90262500	-0.88886000
O	-1.73623500	-5.49636400	-0.04240900
H	-2.20405200	-5.35944400	-0.87602600
H	-2.03062900	-5.48311900	2.50275800
C	7.88817600	1.69089500	1.27086300
H	8.25265100	1.50185000	0.25843900
H	8.17809300	0.84546900	1.90146300
C	8.51679500	2.93022300	1.87397500
H	9.59270900	2.76924200	1.93936200
H	8.12859700	3.10940900	2.87622100
C	-6.02572700	3.19189400	1.14172200
H	-6.49878800	2.25229400	1.43144000
H	-6.18883600	3.91219000	1.94029200
C	-6.52335600	3.73257000	-0.20214400
H	-6.01409000	4.67206100	-0.41498100
H	-6.30723400	3.03135600	-1.00795200
N	8.31584200	4.21845400	1.09648500
C	8.63547800	4.11980200	-0.39907200
C	6.95507300	4.86698200	1.36542800
C	7.36035900	4.00103400	-1.22173300
H	9.31325500	3.27957200	-0.53172200
C	6.66062200	5.91761500	0.30631400
H	6.21932100	4.06874100	1.35611300
C	6.49196500	5.27093500	-1.08627600
H	7.64616500	3.82973500	-2.26084000
H	5.75506200	6.44995600	0.60232700
H	6.76466100	5.99602600	-1.85597100
N	-8.00397100	4.01106000	-0.25066000
C	-8.85310100	2.74170600	-0.33703500
C	-8.50122300	4.93864900	0.84039400
C	-10.30288800	3.01178300	0.08248400
H	-8.38792100	1.99468900	0.30112800
C	-9.88820100	5.47922700	0.46695500
H	-8.52587200	4.35204300	1.75715300
C	-10.73317900	4.43210800	-0.29697100
H	-10.93424700	2.26556300	-0.40236200
H	-10.38098600	5.76329300	1.39878700
H	-10.61991600	4.56975800	-1.37747000
H	9.00656700	4.86323300	1.48119300
H	-8.14706300	4.50970100	-1.13115200
H	7.01347200	5.28329500	2.36974200
H	7.46463200	6.65986400	0.29865900
H	5.44537000	5.00586600	-1.25044300
H	6.79824400	3.12094800	-0.90463600
H	9.17836700	5.02883100	-0.65472500
H	-8.78056200	2.41271700	-1.37272700
H	-10.41176000	2.86809200	1.16052400
H	-11.79158700	4.57724000	-0.07881500
H	-9.78176200	6.38769000	-0.12952000
H	-7.76729200	5.73474400	0.95336800

Pt-Salph+Hasc_betaC_TS

Atom	*X*	*Y*	*Z*
Pt	0.66310900	-0.39774600	-0.77733000
Cl	1.02684600	0.84231700	-3.32472100
Cl	0.22543200	-1.69167300	1.39828000
C	-0.24978900	-2.88299300	3.41209000
O	-2.63331300	-2.49438400	3.35571100
O	-0.61144700	1.08284400	-0.15715300
O	2.18864600	0.70753600	0.02480400
C	-2.66341700	1.98167000	0.42123300
C	-2.63160000	-0.15381700	-0.76221800
C	-4.76317400	0.86254500	-0.08855800
C	1.11493900	-2.93963700	-2.02430200
C	-1.08014500	-3.74138400	-2.69509500
C	-4.04881200	-0.14848200	-0.68353100
C	-1.91056700	0.95065300	-0.17450300
C	-0.28186000	-2.75101700	-2.11307200
C	-2.05679300	-1.28153200	-1.40988900
C	-4.04704300	1.94732500	0.46705500
C	1.68409400	-4.11682100	-2.52079100
C	-0.50089600	-4.90597500	-3.17851500
C	3.13831000	-1.90818800	-1.20425100
C	3.43446800	0.31732400	0.01697400
C	0.88054400	-5.09235700	-3.09435100
C	3.92560300	-0.91423000	-0.55765800
C	4.37259300	1.18263300	0.61430100
C	5.31763000	-1.18268200	-0.47859800
C	5.72161500	0.87686400	0.66047600
C	6.21595800	-0.32656900	0.11060300
H	7.26391000	-0.58390900	0.14856100
H	-2.76562800	-2.01379700	-1.77861200
H	-4.58403600	-0.99231700	-1.10364000
H	-2.13805700	2.81890200	0.86153400
H	-5.84033800	0.80399900	-0.04999200
H	-2.15260400	-3.62201200	-2.74840100
H	2.75082200	-4.28277200	-2.46200800
H	-1.12898200	-5.67001800	-3.61941300
H	3.68954600	-2.76857600	-1.57037500
H	1.33305900	-6.00018100	-3.47358500
H	4.01891000	2.11149400	1.04221600
H	5.68489000	-2.10964900	-0.90463500
O	6.51168700	1.80986800	1.26153400
O	-4.62850000	3.01248800	1.08920100
N	-0.78630900	-1.54784800	-1.55095100
N	1.84759500	-1.89444300	-1.39847400
C	-1.62752600	-3.11839500	3.05104100
C	-1.60343700	-4.40024500	2.20196000
O	-0.22478100	-4.87003000	2.24200000
O	1.67899500	-4.30543300	3.32629600
C	0.53241700	-4.05687800	3.02603400
O	0.12865100	-2.08293300	4.42383600
H	1.07770300	-2.21726700	4.57366900
C	-2.06018900	-4.26724400	0.75458400
H	-1.36926000	-3.59495300	0.23874700
C	-3.48098900	-3.71555900	0.64503700
H	-4.16439500	-4.34034500	1.22860200
H	-3.52961400	-2.68883200	1.01518000
O	-3.81632900	-3.77495900	-0.75236000
H	-4.77368400	-3.81456200	-0.84050400
O	-1.99518800	-5.56912000	0.17753300
H	-2.45664500	-5.49967600	-0.66856800
H	-2.21376800	-5.15916700	2.69988000
C	7.93228100	1.65692300	1.23649400
H	8.28041800	1.48346900	0.21545400
H	8.23866700	0.80398800	1.84944400
C	8.56672600	2.89151600	1.84349600
H	9.64384300	2.73331600	1.89388900
H	8.19063000	3.06101500	2.85204200

C	-6.04872600	3.14561200	1.14150500
H	-6.52898000	2.19859400	1.39359000
H	-6.22943300	3.84272000	1.95691600
C	-6.52044800	3.72575500	-0.19548200
H	-6.00761900	4.67102800	-0.37103600
H	-6.28909100	3.04870100	-1.01762000
N	8.35236500	4.18597200	1.07969000
C	8.65644600	4.10081700	-0.41991400
C	6.99159900	4.82622400	1.36790100
C	7.37283800	3.98266300	-1.22944900
H	9.33663500	3.26479300	-0.56649500
C	6.68172700	5.88436000	0.32065900
H	6.25953100	4.02455600	1.35905200
C	6.50133000	5.24821800	-1.07535000
H	7.64762000	3.81978600	-2.27286600
H	5.77702400	6.41031400	0.63038000
H	6.76370600	5.98035600	-1.84197600
N	-8.00031900	4.00617100	-0.26332000
C	-8.84795300	2.73961200	-0.39255300
C	-8.51639900	4.90761200	0.84072900
C	-10.30511600	3.00092100	0.00687200
H	-8.39437200	1.97850400	0.23744000
C	-9.89643500	5.45822700	0.45654600
H	-8.55773800	4.29882300	1.74221200
C	-10.72833800	4.43008400	-0.34652600
H	-10.92789400	2.26679500	-0.50670200
H	-10.40511500	5.72030900	1.38629300
H	-10.59621300	4.59306200	-1.42128700
H	9.04448400	4.83038500	1.46256200
H	-8.12858000	4.52442900	-1.13475100
H	7.05827800	5.23421300	2.37513400
H	7.48239700	6.63023000	0.31054200
H	5.45412700	4.98058000	-1.23116400
H	6.81742300	3.09828400	-0.91225300
H	9.19231200	5.01441600	-0.67403800
H	-8.75700400	2.43338600	-1.43361500
H	-10.43351100	2.83204800	1.07909500
H	-11.79037700	4.57072500	-0.14352900
H	-9.77939800	6.38063400	-0.11607500
H	-7.78432900	5.70007000	0.98618400

Pt-Salph+Hasc_betaC_PROD

Atom	*X*	*Y*	*Z*
Pt	-0.83857100	-0.28767900	0.59112100
Cl	-1.36263400	0.80775000	4.39659500
Cl	0.38743300	-2.43971200	-1.81224800
O	3.47822300	-3.01191500	-3.09219700
C	2.60428900	-3.69636100	-2.63979700
O	0.35003400	1.27188900	-0.00875400
O	-2.43392400	0.72895200	-0.19198500
C	2.34812700	2.33736900	-0.50246700
C	2.43559600	0.19484900	0.66792500
C	4.50640100	1.37885200	0.08008800
C	-1.13739900	-2.85043000	1.83530400
C	1.08325600	-3.46240600	2.61335400
C	3.84973100	0.31322000	0.64749100
C	1.65285000	1.24681700	0.06081600
C	0.23919700	-2.55908200	1.95946400
C	1.92262000	-0.97918500	1.28755200
C	3.73037800	2.40693300	-0.50276300
C	-1.63385000	-4.05605300	2.34025200
C	0.57571300	-4.65604300	3.10867900
C	-3.21099100	-1.95980500	0.98857500
C	-3.65149100	0.25986900	-0.20043500
C	-0.78108300	-4.95400900	2.96929900
C	-4.06206200	-1.01282900	0.34971800
C	-4.64493100	1.06986400	-0.78992000
C	-5.43269400	-1.37143900	0.25871600
C	-5.96826600	0.67275600	-0.85411700
C	-6.38389600	-0.56857700	-0.32409500
H	-7.41233200	-0.89445100	-0.37324100
H	2.67115900	-1.64738900	1.70045100
H	4.43167000	-0.48439800	1.09549300
H	1.77662800	3.13434400	-0.96017500
H	5.58551000	1.40447800	0.08764900
H	2.13728700	-3.24931400	2.72502000
H	-2.68236500	-4.30412900	2.24976500
H	1.24041400	-5.35316400	3.60339300
H	-3.70484500	-2.85684900	1.34854400
H	-1.17797200	-5.88491700	3.35509600
H	-4.35235100	2.02773700	-1.20012200
H	-5.73963500	-2.32745600	0.66858200
O	-6.81654700	1.55611800	-1.45553100
O	4.25136600	3.51643800	-1.10529200
N	0.66665100	-1.33624000	1.37242900
N	-1.92133200	-1.85712500	1.18178200
C	1.12942600	-3.67778600	-3.02093900
C	2.75434100	-4.80651900	-1.60028500
O	1.51516400	-5.57339600	-1.68240100
O	-0.40540400	-5.55367000	-2.85581700
C	0.62247100	-5.03400900	-2.52993700
O	0.87197200	-3.37547400	-4.31024800
H	-0.00908000	-3.71139200	-4.54023300
C	2.96509700	-4.33225400	-0.16444900
H	2.07947600	-3.76083300	0.13782400
C	4.20493400	-3.45057200	-0.01731100
H	5.08770600	-3.99204100	-0.36921600
H	4.10047400	-2.52305400	-0.58735200
O	4.30567500	-3.17367900	1.38708200
H	5.22735300	-3.01262800	1.61293500
O	3.08912800	-5.50079600	0.62927500
H	3.43204000	-5.20558100	1.48337900
H	3.56497100	-5.47582800	-1.89317600
C	-8.22514000	1.32449000	-1.41345300
H	-8.55191400	1.13820000	-0.38732200
H	-8.49237700	0.45252100	-2.01810800
C	-8.93260800	2.51913000	-2.02058000
H	-10.00106600	2.30651000	-2.05127800
H	-8.58178900	2.69741600	-3.03676000
C	5.66188400	3.71570200	-1.17743400
H	6.18235800	2.78947500	-1.42794400
H	5.80161600	4.41369500	-2.00044900
C	6.12645200	4.32840500	0.14741000
H	5.59058800	5.26228800	0.31460100
H	5.91956200	3.65739600	0.98079100
N	-8.77097900	3.83047800	-1.27239000
C	-9.04370300	3.74425100	0.23269300
C	-7.44931400	4.53577700	-1.59081200
C	-7.74262000	3.68594300	1.02087500
H	-9.68582100	2.88180700	0.39689700
C	-7.16805600	5.60882800	-0.55075300
H	-6.67953400	3.77031400	-1.59726200
C	-6.92445300	4.98360700	0.83998300
H	-7.99363200	3.52301900	2.07025400
H	-6.29793100	6.17895000	-0.88083300
H	-7.19185800	5.70770200	1.61245200
N	7.59955400	4.64694400	0.19795600
C	8.47866300	3.40296200	0.33755500
C	8.08368500	5.54257300	-0.92508600
C	9.92549900	3.69295700	-0.07952500
H	8.03764400	2.62219100	-0.27723300
C	9.45319200	6.13255400	-0.56250000
H	8.13226300	4.92034200	-1.81689800
C	10.31710000	5.13720600	0.24804200
H	10.57049300	2.98220800	0.43944900
H	9.94681200	6.39266000	-1.50090300
H	10.19082100	5.31309900	1.32143300
H	-9.50163100	4.43569800	-1.64823900
H	7.72337700	5.18171500	1.06004900
H	-7.55768600	4.93908300	-2.59645500
H	-8.00466700	6.31352500	-0.52126300
H	-5.86382400	4.75602900	0.96590100
H	-7.15778200	2.82134900	0.70282000
H	-9.61261800	4.63682300	0.48941100
H	8.40440100	3.10970100	1.38367600
H	10.04845900	3.51049300	-1.15015500
H	11.37352500	5.30029700	0.03291000
H	9.31870500	7.06050700	-0.00280200
H	7.33151200	6.31466900	-1.07700000
